# Hypoxia-induced lncRNA RBM5-AS1 promotes tumorigenesis via activating Wnt/β-catenin signaling in breast cancer

**DOI:** 10.1038/s41419-022-04536-y

**Published:** 2022-02-02

**Authors:** Xinping Li, Jingyan Yang, Ruiqi Ni, Jintian Chen, Yanhao Zhou, Hao Song, Liang Jin, Yi Pan

**Affiliations:** State Key Laboratory of Natural Medicines, Jiangsu Key Laboratory of Druggability of Biopharmaceuticals, School of Life Science and Technology, China Pharmaceutical University, 24 Tongjiaxiang Avenue, Nanjing, Jiangsu Province China

**Keywords:** Breast cancer, Cancer stem cells, Long non-coding RNAs

## Abstract

Breast cancer is the most common malignancy among women across the globe. Recent studies have revealed that many long non-coding RNAs (lncRNAs) regulate the Wnt/β-catenin signaling pathway in several types of cancer. Hyperactivation of the Wnt/β-catenin pathway has been extensively presented in breast cancer and is involved in breast cancer progression. However, the underlying molecular mechanism remains elusive. In the current study, we found lncRNA RBM5-AS1 was remarkably upregulated in breast cancer cells and tissues. Overexpression of RBM5-AS1 facilitated proliferation, migration, invasion, EMT, and stemness maintenance of breast cancer cells in vitro and in vivo. Mechanism studies suggested that RBM5-AS1 could be transcriptionally activated by hypoxia-induced RUNX2. Upregulated RBM5-AS1 further activated the Wnt/β-catenin signaling by preventing β-catenin degradation and by helping organize β-catenin-TCF4 transcriptional complex. These findings suggested that RBM5-AS1, a regulator of Wnt/β-catenin signaling, plays a vital role in breast cancer initiation and progression, implicating its potential as a new target for breast cancer treatment.

## Introduction

Breast cancer is the most common cancer and the predominant cause of tumor-related deaths among females [1]. Despite advancements in diagnostic, surgery, and numerous anticancer treatments, the prognosis of patients with breast cancer is not satisfactory. Breast cancer is a heterogeneous disease originated from the breast ductal epithelium, with carrying complex genetic, epigenetic, and environmental factors between individuals [2]. As a result, it is urgent to identify novel pathogenic genes and carcinogenic pathways underlying breast cancer initiation and progression.

The excessive activation of the Wnt/β-catenin pathway serves a critical role in breast cancer cell growth, survival, apoptosis, invasion, migration, epithelial-mesenchymal transition (EMT), and stemness maintenance [3]. When the Wnt signaling is inhibited, cytosolic β-catenin is normally sequestrated in the destruction complex consisting of AXIN1, APC, GSK-3β, and CK1, and then gets degraded via the ubiquitin-proteasome system. Upon Wnt activation, disruption of the destruction complex leads to the cytoplasmic β-catenin accumulation and its subsequent nuclear translocation [4]. Nuclear β-catenin could form transcription complexes with transcription factors like T-cell factor 4 (TCF4) then initiate the transcription of downstream target genes [5]. Wnt/β-catenin signaling is also essential for the generation and maintenance of breast cancer stem cells (BCSCs), which is the major cause of tumorigenesis, metastasis, and relapse [6], and has been found to be associated with worse outcomes in breast cancer patients as well [7]. Hence, targeting the Wnt signaling has been applied as a novel therapeutic strategy for the treatment of breast cancer.

Long non-coding RNAs (lncRNAs) are non-coding transcripts with lengths exceeding 200nt. Dysregulation of lncRNAs have been discovered to be involved in tumorigenesis and development in diverse cancers [6]. They modulate gene expression via epigenetic modification, transcriptional and post-transcriptional regulation. It is worth mentioning that lncRNAs exert different vital functions in breast cancer development. For instance, hypoxic lncRNA KB-1980E6.3 maintains BCSCs stemness through interacting with IGF2BP1 and stabilizing c-Myc mRNA [8], lncRNA CCAT1 is crucial for breast cancer occurrence and metastasis via regulating BCSCs [6]. However, the clinical implications and molecular mechanisms of lncRNAs regulating breast cancer stemness through Wnt/β-catenin pathway, remain largely unknown.

Our previous lncRNA microarray analysis identified that RBM5-AS1 (RBM5 antisense RNA 1) was upregulated in both BCSCs MCF-7 cells (CD44+CD24−) and poorly differentiated breast cancer tissues (high-grade) compared with non-BCSCs MCF-7 cells (non-CD44+CD24−) and paracancerous normal tissues [6]. According to previous studies, RBM5-AS1 promotes self-renewal properties of colon cancer stem cells [9], and exerts pro-oncogenic effects in osteosarcoma, hepatoma, and oral squamous cell carcinoma [10–12]. Nevertheless, the biological role of RBM5-AS1 in breast cancer is still far from clear.

In the current work, we attempted to decipher the regulatory function and mechanisms of RBM5-AS1 in breast cancer cells. Our data revealed that hypoxia-induced RBM5-AS1 activates Wnt/β-catenin signaling pathway through upregulating as well as binding β-catenin to facilitate breast cancer initiation and progression, with implications of RBM5-AS1 as a promising therapeutic target for breast cancer.

## Materials and methods

### Clinical samples from patients

We obtained patient samples from Jiangsu Provincial Breast Disease Center, First Affiliated Hospital of Nanjing Medical University (Nanjing, China), all the specimen collection informed patients and had their written consent. The Ethical Review Committees in China Pharmaceutical University (Nanjing, China) ethically approved our study, which was performed in accordance with the Helsinki Principles Declaration. Reviewed by practiced clinical pathologists, tumor sample histology and biopsy were used as the basis for pathological diagnosis. Pathological characteristics of the clinical samples can be found in supplementary documents (Table S1) and the breast cancer or paracancerous normal tissues were snap-frozen in liquid nitrogen until required.

### Cell lines culture and sphere-forming assay

In our study, cell lines including breast cancer cell lines, embryonic kidney cell line, and the non-tumorigenic epithelial breast cell line were cultured and employed. The cell lines were purchased from the Chinese Academy of Sciences (Shanghai, China) and then stored at 37 °C with 5% CO_2_ in a humidified incubator. DMEM medium (Gibco) with 10% fetal bovine serum (FBS; Gibco) was used to culture MCF-7, T47D, SKBR-3 MDA-MB-231, MDA-MB-468 cells, and the embryonic kidney cell line (HEK-293T). BT-549 cells were grown in RPMI-1640 medium (Gibco) with 10% FBS (Gibco). DMEM/Ham’s F-12 (1:1; Gibco) with 5% FBS (Gibco) was used to culture noncancerous mammary epithelial breast cell line, MCF-10A. Cell lines were all authenticated recently and they showed negative in mycoplasma contamination test. Sphere formation assay was performed as described before [6], cells were cultured in low-cell adhesion plates (Corning) containing 24 wells, each of which contains DMEM/F12 (Invitrogen) without serum. Besides, they were supplemented with 1% double-antibiotics (Biyuntian), 20 ng/ml EGF (Peprotech) and bFGF (Invitrogen), 20% methylcellulose (Sigma), 4 µg/ml insulin (Sigma), and B27 (1:50, Invitrogen). Lasting for two weeks, cells were cultured in a CO_2_ incubator, and a stereomicroscope (Olympus) was used to count spheroid cells number.

### Hypoxic culture

To induce the hypoxic condition, a mixture of gas containing 1% O_2_ (with 94% N_2_ and 5% CO_2_) was pumped into a hypoxic chamber (Invivo2 400 hypoxia workstation; Ruskinn technology), and cells were cultured in the hypoxic chamber (BioSpherix) for 24 or 48 h.

### RNA extraction and real-time quantitative polymerase chain reaction (RT-qPCR)

Total RNA was extracted from breast cancer cells or clinical tissue samples using RNeasy kit (QIAGEN) in accordance with the instructions of manufacturers. Then, isolated RNA was reversely transcribed to cDNA using PrimeScript^TM^ RT reagent Kit (Takara), and RT-qPCR was performed using the SYBR Premix Ex Taq II Kit (Takara). Primers used here can be found in Table S2. The mRNA expression was quantified by referenced against GAPDH with the 2^−ΔΔCt^ method.

### Plasmid constructions

Using Super-Fidelity DNA Polymerase (Vazyme), the cDNAs of RBM5-AS1, pGL3-Basic-RBM5-AS1 pro-WT, and RUNX2 were amplified and they were cloned into pcDNA3.1 (Invitrogen). Using primers named S1-S4 respectively, four RBM5-AS1 fragments carrying deletions for RNA pull down assays recruited pcDNA3.1-RBM5-AS1 as a model to produce constructs. The pGL3-Basic-RBM5-AS1 pro-WT with point mutations in the RUNX2 response elements was synthesized by Mut Express^®^ II Fast Mutagenesis Kit V2 System (Vazyme) and named as pGL3-Basic-RBM5-AS1 pro-MUT. DNA sequencing was used to verify all PCR products and primers employed for constructing plasmid can be found in Table S2 as well.

### Knockout by siRNAs or anti-sense nucleotides (ASO)

As a pool comprising three siRNAs and three anti-sense oligo-nucleotides, RiboTM LncRNA Smart Silencer (Ribobio) was adopted for transient knockdown of RUNX2, lncRNA RBM5-AS1, CTCF, and CTNNB1 (β-catenin mRNA), while RBM5-AS1 ASO (Ribobio) was also utilized in this process.

### Western blot assay

To separate cell lysates, SDS-PAGE electrophoresis was carried out and the target proteins were immunoblotted by primary antibodies and secondary antibodies, and then visualized by an enhanced chemiluminescence kit (Tanon), Tanon 5200 scanner and GelCap software (Tanon). All the antibodies used for western blot assay can be found in Table S3, and the uncropped blots are provided in Supplementary Data.

### Proliferation, migration, and invasion assay

As described before [13], plate colony assay was carried out to measure the capacity of cell proliferation. Also, the Cell Counting Kit-8 (CCK-8) assay using CCK-8 (Dojindo Laboratories) and the 5-Ethynyl-2’-deoxyuridine (EdU) assay using EdU Assay Kit (RiboBio) were performed to assess cell proliferation capability under the instructions of manufacturers. EdU positive cells ratio was recorded under fluorescence microscope (Zeiss). As for analyzing cell migration and invasion abilities, we did scrape motility assay and transwell assay based on the published method [6].

### Flow cytometry

Cells were labeled by corresponding antibodies following instructions of the manufacturer to be detected using a FACSCalibur (BD Biosciences) as described [14]. To implement cell sorting, MCF-7 cells or MDA-MB-231 cells were incubated with cocktail anti-human CD24 antibody conjugated with APC and anti-human CD44 antibody conjugated with PE. Then, they were sorted by FACS Aria III (BD). All the antibodies for flow cytometric analyses are shown in Table S3.

### TOP/FOP-Flash reporter luciferase assay

Cells were transfected with TOP-FLASH or FOP-FLASH reporter plasmid, plus Renilla luciferase vector. Forty-eight hours later, Dual-Luciferase Reporter Assay System (Promega) was applied to measure luciferase activity. Then, TOP or FOP values were normalized to Renilla values. The calculated TOP/FOP ratios were used to indicate the endogenous intensity of WNT signaling.

### Coimmunoprecipitation (co-IP) assay

Using IP lysis buffer (Biyuntian) to lyse the cells. Then, the cell lysates were incubated with a mixture of 40 ml protein-A/G Agarose beads (Millipore), β-catenin antibody, and TCF-4 antibody (Cell Signaling Technology) at 4 degrees centigrade overnight. Using RIPA buffer to wash three times, the samples were then subjected to SDS-PAGE.

### Chromatin immunoprecipitation (ChIP)

ChIP assay was implemented by EZ-ChIP^™^ Kit (Millipore). The chromatin immunoprecipitated with antibodies was analyzed by quantitative PCR. By 1% formaldehyde, chromatin-immunoprecipitated cells were cross-linked or transfected. Then, the DNA was cut to an average 400 bp fragmentations, before they were immunoprecipitated by antibody against RUNX2 (anti-RUNX2, Abcam). To amplify promoter regions of RBM5-AS1 which contain putative RUNX2 binding sites, the primers for ChIP-PCR were designed specifically. The immunoprecipitated DNA was then washed and eluted. Furthermore, the specific PCR primers were employed to measure level of RUNX2 as well as RBM5-AS1 promoter fragments existing in the immunoprecipitated DNA. We calculated the fold-enrichment (FE) by the formula: FE% = 2 (IgG CT-Sample CT) 100%. RNA Polymerase II amplification-efficiency was regarded as a positive control. The primer sequences can be found in Table S2.

### Animal experiments

We purchased female BALB/c nude mice aged 5–6 weeks (18–20 g) from the Model Animal Research Center of Nanjing University (Nanjing, China). The mice were taken care of in accordance with Provisions and General Recommendation of Chinese Experimental Animals Administration Legislation, and all animal experiments procedure was under IACUC (Institutional Animal Care and Use Committee) regulations. Control lentivirus, RBM5-AS1 lentivirus, control ASO and RBM5-AS1 ASO were stably transfected into MCF-7 cells. In vivo limiting dilution assay was implemented to assess the ability of tumor formation. 1 × 10^4^, 1 × 10^5^, and 1 × 10^6^ indicated MCF-7 cells were injected into female BALB/c nude mice which were five-week-old (*n* = 8/group) before frequency of tumor-initiating was calculated. Twenty-four days after injection, mice were sacrificed by injecting with sodium pentobarbital (150 mg/kg, Sigma) for euthanasia to reduce suffering and distress to the minimum according to AVMA Guidelines for the Euthanasia of Animals. Then, the subcutaneous tumors were peeled and the tumor size and volume were measured. Then the paraffin-embedded tissues were sectioned for hematoxylin-eosin(H&E) staining and immunohistochemical (IHC) analysis of Ki67, ALDH1A1, Oct4, Nanog, Sox2, CD44, c-Myc, c-Jun, Cyclin D1, MMP7, and AXIN1, which were entrusted to Servicebio (Wuhan, China). The used antibodies are provided in Table S3. The investigators and operators were blinded to group allocation with regard to all animal experiments, which gained approval from the Ethics Committee at China Pharmaceutical University (Permit Number: x2162326).

### Statistics and analysis

Data visualization, as well as statistical analyses, were implemented in virtue of Prism 8 (GraphPad). Student’s *t*-test and Pearson correlation analysis were applied for two-group comparisons and group correlation analysis, respectively. For all analyses, *P*-value of < 0.05 was considered statistically significant. Unless stated differently, experiments were repeated independently at least three times with at least three replicates, and data were shown as the mean ± SEM. The legends prompt details about specific applied tests. All statistics were calculated and obtained from *n* = 3 technical replicates from a single experiment (unless otherwise noted below). Variance is similar across the groups. Data are reported as mean ± SEM.

## Results

### RBM5-AS1 is significantly upregulated in breast cancer tissues and BCSCs

**Fig. 1:**
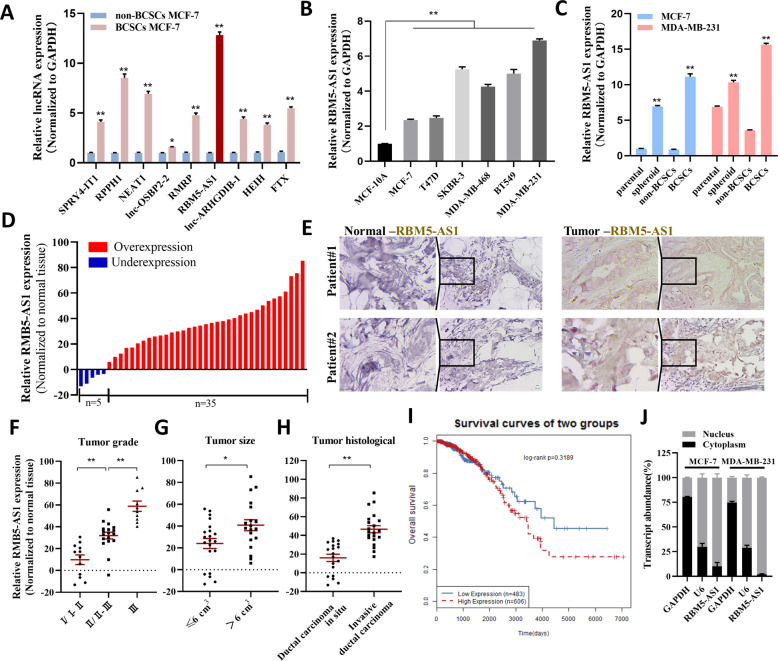
Expression of RBM5-AS1 in breast cancer tissues and BCSCs. **A** Verification of the significantly upregulated (>2.0-fold) lncRNAs in BCSC MCF-7 cells compared to non-BCSC MCF-7 cells detected by lncRNA microarrays. RBM5-AS1 levels examined in diverse breast cancer cell lines (**B**) and BCSCs (**C**). **D** RBM5-AS1 levels examined in 40 paired breast cancer tissues and paracancerous normal tissues by RT-qPCR. **E** RBM5-AS1 expression levels in breast cancer tissues (Tumor) and paracancerous normal tissues (Normal) were visualized by ISH. Representative ISH images from patients #1 and #2 are shown. Scale bar, 25 μm. RBM5-AS1 levels in breast cancer patients with different pathological grades (**F**), tumor sizes (**G**), and tumor histological types (**H**). **I** The correlation of RBM5-AS1 levels and overall survival (OS) in breast cancer patients from LncACTdb database. **J** RBM5-AS1 distribution in breast cancer cells was examined by nuclear-cytoplasm separation of MCF-7 and MDA-MB-231 cells and RT-qPCR. GAPDH and U6 served as internal controls. All data are shown as the mean ± SEM. *P < 0.05 and **P < 0.01 by two-tailed Student’s *t*-test.

Our previous study has identified 10 lncRNAs remarkably upregulated in both BCSCs MCF-7 cells and poorly differentiated breast cancer tissues compared with non-BCSCs MCF-7 cells and paracancerous normal tissues [6]. We first confirmed that these genes, except lncRNA CCAT1 which we have studied previously [6], were upregulated in BCSCs MCF-7 cells. As shown in Fig. 1A, RBM5-AS1, as the most substantially upregulated lncRNA, particularly caught our attention. To evaluate the relevance of RBM5-AS1 in breast cancer progression, we further assessed the expression of RBM5-AS1 in multiple breast cancer cell lines and BCSCs. We could observe significantly higher RBM5-AS1 levels in diverse breast cancer cell lines than in normal mammary epithelial MCF-10A cells (Fig. 1B). We employed MCF-7 and MDA-MB-231 cells which had different RBM5-AS1 expression pattern for further study. As shown, RBM5-AS1 levels were also increased in MCF-7 and MDA-MB-231 spheroid cells that enriched in BCSCs, sorted BCSCs MCF-7 cells (CD44+CD24−) and BCSCs MDA-MB-231 cells (CD44+CD24-ESA+), compared to adherent cells and non-BCSCs, respectively (Fig. 1C). Besides, the upregulation of RBM5-AS1 was confirmed in breast cancer tissues (Fig. 1D). Remarkably, RBM5-AS1 expression was upregulated in 87.5% (35 of 40 paired) of the breast tumor tissues. ISH assays also indicated that RBM5-AS1 was expressed in breast tumor cells, whereas little signal was observed in the normal breast tissues (Fig. 1E). We further divided the samples into low (*n* = 20) and high (*n* = 20) RBM5-AS1 expression groups and analyzed the relevance between the clinicopathological characteristics and RBM5-AS1 levels in breast cancer patients. As Table S1 and Fig. 1F–H shown, we found a positive association between RBM5-AS1 levels and tumor grade, tumor histological, and tumor size in this study. Moreover, we assessed the relevance of RBM5-AS1 expression levels to breast cancer prognosis using LncACTdb (http://www.bio-bigdata.net/lncactdb/) based on TCGA database. Although there was no significant difference of the overall survival between the RBM5-AS1 low and high expression groups, we found higher expression RBM5-AS1 indeed portended a worse outcome in breast cancer (Fig. 1I). We also found that RBM5-AS1 located predominantly in the nucleus of breast cancer cells (Fig. 1J), consistent with a prior study [9]. Collectively, the above data demonstrated the upregulations of RBM5-AS1 in breast cancer cells and tissues.

### Hypoxia induces the transcription of RBM5-AS1 through upregulating RUNX2

**Fig. 2:**
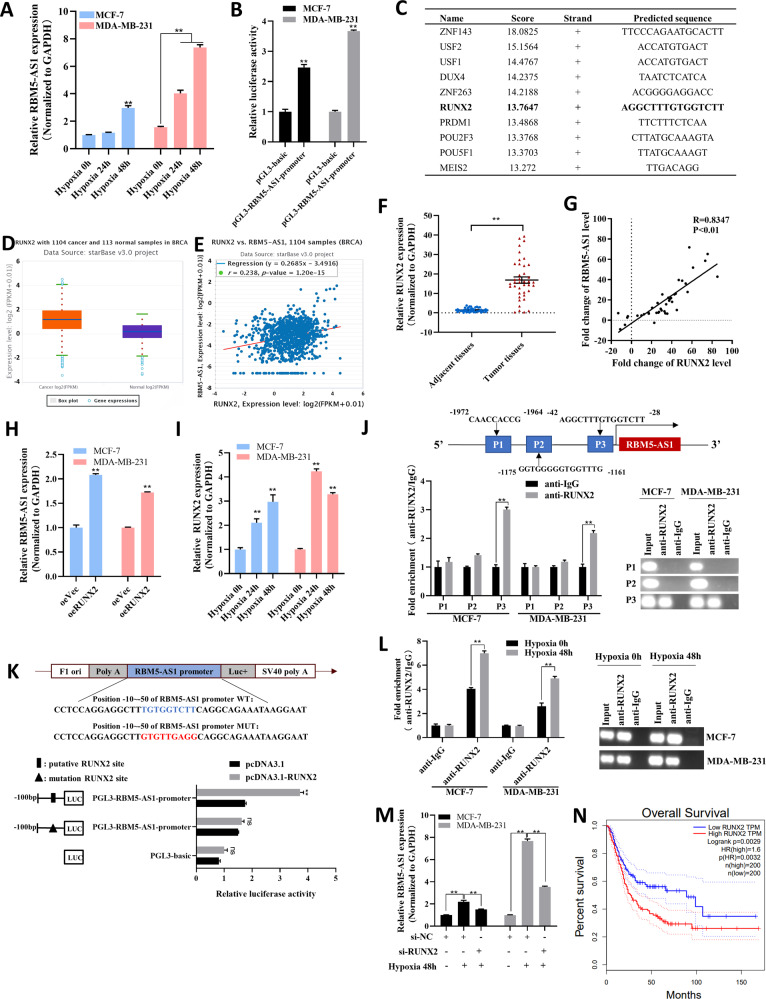
Hypoxic-induced RUNX2 upregulates RBM5-AS1 expression in breast cancer cells. **A** RBM5-AS1 levels in MCF-7 and MDA-MB-231 cells under hypoxic condition for 24 and 48 h. **B** Transcriptional activity of the RBM5-AS1 promoter region. The pGL3-RBM5-AS1-promoter were transfected into MCF-7 and MDA-MB-231 cells, then luciferase activity was checked. **C** Binding sequences of top-10 ranked transcription factors on RBM5-AS1 promoter predicted in JASPAR website. **D** RUNX2 levels in 1104 breast cancer tissues (left) and 113 normal breast tissues (right) from TCGA database extracted using StarBase 3.0. **E** Spearman correlation analysis of the RUNX2 mRNA and RBM5-AS1 levels in 1104 human breast cancer tissues from TCGA project extracted using StarBase 3.0. **F** RUNX2 levels in 40 paired breast cancer tissues and paracancerous normal tissues. **G** Spearman correlation analysis of the correlation between RUNX2 and RBM5-AS1 levels in 40 paired breast cancer tissues. (**H**) RBM5-AS1 levels in control or RUNX2-overexpressing MCF-7 and MDA-MB-231 cells. (**I**) RUNX2 levels in MCF-7 and MDA-MB-231 cells under hypoxic condition for 24 and 48 h. **J** Putative RUNX2 binding sites on the promoter region of RBM5-AS1 (upper). Enrichment of RUNX2 on RBM5-AS1 promoter in MCF-7 and MDA-MB-231 cells detected by ChIP assays (lower left) and PCR analysis (lower right). Approximately one −42 to −28 bp fragment of the RBM5-AS1 promoter is responsible for RUNX2 binding. **K** Mutagenesis of the putative RUNX2 binding site (−50 to −10 bp) on the RBM5-AS1 promoter abolished the transcriptional activation of RBM5-AS1 by RUNX2 in MCF-7 cells. **L** Enrichment of RUNX2 on RBM5-AS1 promoter region in MCF-7 and MDA-MB-231 cells under hypoxic condition for 48 h detected by ChIP-qPCR assays (left) and PCR analysis (right). **M** RBM5-AS1 levels in si-NC or si-RUNX2 transfected MCF-7 and MDA-MB-231 cells under hypoxic condition for 0 or 48 h. **N** The relationship between RUNX2 expression and OS of breast cancer patients from GEPIA website. All data are shown as the mean ± SEM. *P < 0.05 and **P < 0.01 by two-tailed Student’s *t*-test.

Next, we investigated the underlying mechanisms of RBM5-AS1 upregulation in breast cancer. Given that hypoxia is a crucial microenvironmental factor contributed to tumorigenesis, as well as maintenance and self-renewal of cancer stem cells [14], we tested whether RBM5-AS1 upregulation was induced by hypoxia. After exposure to hypoxia condition for 24 h and 48 h, RBM5-AS1 level was increased gradually in MCF-7 and MDA-MB-231 cells (Fig. 2A). To identify the transcriptional factor mediating the hypoxia-induced RBM5-AS1 overexpression, we first used UCSC Genome Browser (https://genome.ucsc.edu) to predict the promoter sequence of RBM5-AS1. Then we constructed the RBM5-AS1 promoter reporter plasmids (pGL3-RBM5-AS1-promoter) and transfected it into MCF-7 and MDA-MB-231 cells to verify the transcriptional activity of the predicted RBM5-AS1 promoter region (Fig. 2B). Next, we consulted JASPAR database to predict the potential transcriptional factors of RBM5-AS1 (Fig. 2C). Among the candidates, we noticed that RUNX2, which was involved in tumor growth, invasion, and metastasis, had been reported to be increased by hypoxia [15]. Furthermore, the upregulation of RUNX2 (Fig. 2D, F), and the positive correlation between RUNX2 and RBM5-AS1 expression (Fig. 2E, G), were validated in breast cancer tissues from StarBase database or collected by us. Also, we confirmed that RBM5-AS1 could be upregulated by RUNX2 overexpression (Fig. 2H), and RUNX2 expression could be induced by hypoxia (Fig. 2I) in breast cancer cells. Subsequently, we predicted three RUNX2 binding sites on the RBM5-AS1 promoter by bioinformatics analysis (Fig. 2J, upper). ChIP assay identified that RUNX2 was enriched in the promoter region within −42 to −28 bp upstream of the transcription start point of RBM5-AS1 (Fig. 2J, lower), which was further confirmed by luciferase reporter assays (Fig. 2K). Moreover, hypoxia enhanced the enrichment of RUNX2 in the RBM5-AS1 promoter region (Fig. 2L), while RUNX2 knockdown (Fig. S1A) abolished the hypoxia-induced RBM5-AS1 upregulation (Fig. 2M). Investigation of the association between RUNX2 expression and breast cancer patient prognosis also revealed that patients had a worse outcome as RUNX2 elevated (Fig. 2N). Taken together, the data presented above indicated that in breast cancer, hypoxia-induced RUNX2 could facilitate RBM5-AS1 transcription.

### RBM5-AS1 is essential for maintaining stemness and promoting growth, migration, and invasion of breast cancer cells

**Fig. 3:**
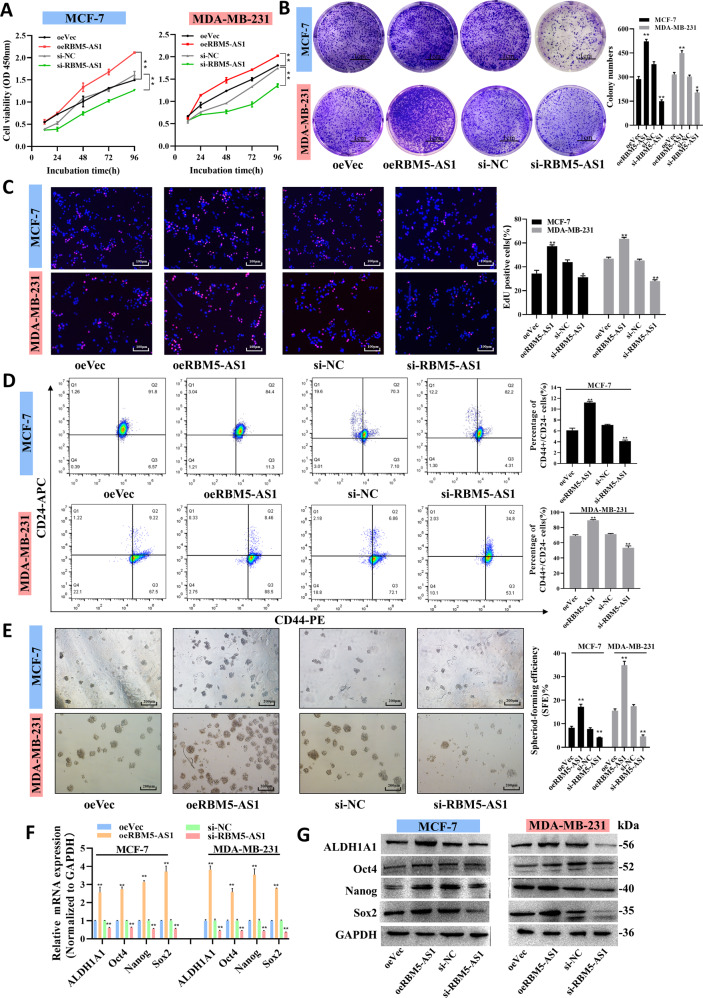
RBM5-AS1 promotes proliferation and stemness of breast cancer cell in vitro. Proliferation capacity of MCF-7 and MDA-MB-231 cells with RBM5-AS1 overexpression or knockdown examined by CCK-8 assays (A), plate colony formation assays (**B**, scale bar, 1 cm) and EdU assays (**C**, scale bar, 100 μm). **D** Proportion of CD44+CD24− cells in RBM5-AS1-overexpressing or RBM5-AS1-knocked down MCF-7 and MDA-MB-231 cells detected by flow cytometric analysis. **E** Sphere-formation abilities of MCF-7 and MDA-MB-231 cells with RBM5-AS1 overexpression or knockdown. SFEs are shown in the right panel. Scale bar, 200 μm. mRNA (**F**) and protein levels (**G**) of pluripotent transcription factors in MCF-7 and MDA-MB-231 cells with RBM5-AS1 overexpression or knockdown. All data are shown as the mean ± SEM. *P < 0.05 and **P < 0.01 by two-tailed Student’s *t*-test.

To uncover the potential role of RBM5-AS1 in breast cancer progression, functional experiments were performed in control, RBM5-AS1-overexpressing or -knocked down MCF-7 and MDA-MB-231 cells (Fig. S1B). To ensure that the observed phenotype is only due to RBM5-AS1 instead of its sense counterparts, we first proved that overexpression or knockdown of RBM5-AS1 would not cause the alternation of RBM5 and RBM6 mRNA and protein levels (Fig. S1C–E). The CCK-8, plate colony formation and EdU incorporation assays revealed that RBM5-AS1 overexpression could significantly facilitate MCF-7 and MDA-MB-231 cell proliferation, whereas RBM5-AS1 depletion inhibited the proliferative capacities of cells (Fig. 3A–C). Flow cytometry analysis indicated that RBM5-AS1 overexpression increased the ratio of CD44+CD24− BCSCs, while RBM5-AS1-knocked down remarkably reduced this proportion in MCF-7 and MDA-MB-231 cells (Fig. 3D). In addition, RBM5-AS1 overexpression elevated the sphere-forming efficiency (SFE), whereas RBM5-AS1-knockdown impaired the capacity for self-renewal in serial passaging (Fig. 3E). Moreover, RBM5-AS1 overexpression and knockdown markedly changed the expression of pluripotency genes ALDH1A1, Oct4, Nanog, and Sox2 (Fig. 3F, G), which play the major role in regulating cell stemness properties, including self-renewal.

We next studied the effects of RBM5-AS1 in modulating breast cancer cell migration and invasion using scratch assays (Fig. S2A–C) and transwell assays (Fig. S2D–F). As shown, the migratory and invasive abilities of cells were remarkably enhanced through overexpressing RBM5-AS1 whereas suppressed by depleting RBM5-AS1. Furthermore, RBM5-AS1 promoted EMT process, which contributes to tumor migration and invasion (Fig. S2G). Overall, the results provided above illustrated that RBM5-AS1 exerts pro-oncogenic effects in breast cancer cell by promoting proliferation, stemness induction and maintenance, migration, and invasion of breast cancer cells.

### RBM5-AS1 enhances tumorigenicity of breast cancer cells in nude mice

**Fig. 4:**
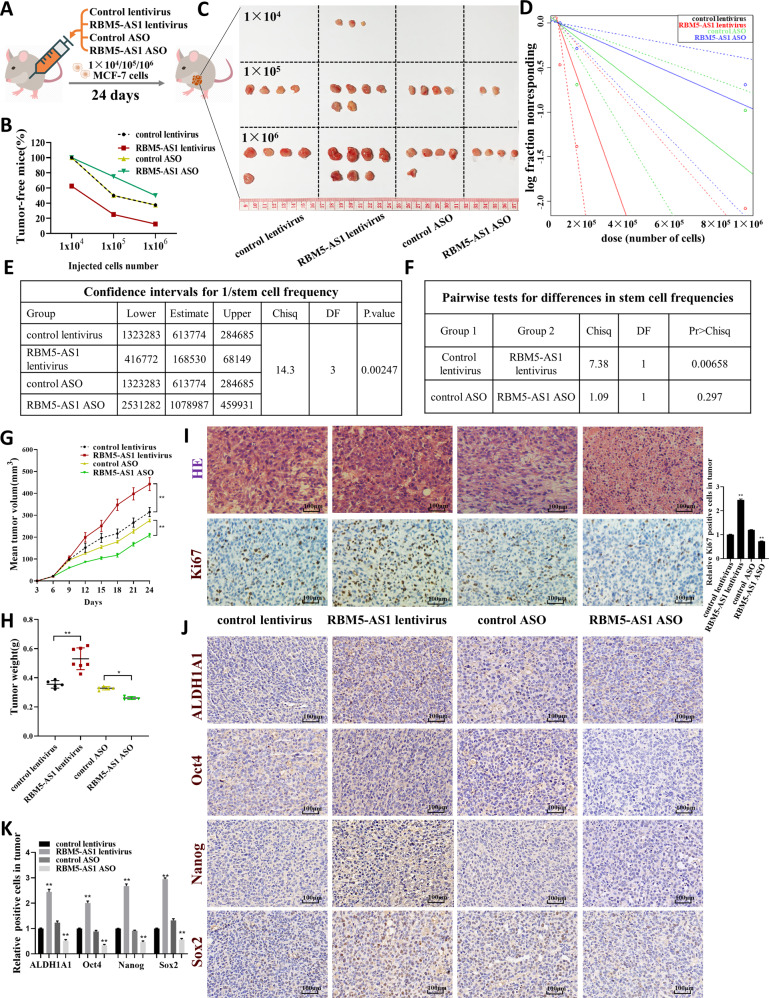
RBM5-AS1 enhances in vivo tumorigenicity of breast cancer cells. **A** Workflow of the in vivo xenograft experiments designed for examining tumor proliferative capacity and stemness. **B** In vivo limiting dilution tumorigenicity studies of indicated MCF-7 cells were used to determine the efficacy of RBM5-AS1 on the tumor-initiating frequency. The xenografts were dissected and measured after 24 days; *n* = 8 for each group. Images (**C**) and index (**D**–**F**) of tumors harvested after different MCF-7 cells serially diluted were planted for 24 days. Tumor volume (**G**) and weight (**H**) of the tumors from mice subcutaneously implanted with 1 × 10^6^ cells were measured. Representative images of HE staining and Ki-67 IHC staining (**I**), ALDH1A1, Oct4, Nanog, and Sox2 IHC staining (**J**) of the tumor sections. Scale bar, 100 μm. IHC staining of ALDH1A1, Oct4, Nanog, and Sox2 were performed three times independently (**K**). All data are shown as the mean ± SEM. *P < 0.05 and **P < 0.01 by two-tailed Student’s *t*-test.

To examine whether RBM5-AS1 participated in breast tumorigenesis in vivo, we conducted in vivo limiting dilution tumor initiation assay (Fig. 4A). MCF-7 cells transfected with control lentivirus, RBM5-AS1 lentivirus, control ASO, or RBM5-AS1 ASO were injected into nude mice via the armpit at a 10-fold dilution series from 1 × 10^6^ to 1 × 10^4^ cells per mouse. We used RBM5-AS1 lentivirus and RBM5-AS1 ASO to overexpress or knockdown RBM5-AS1 in MCF-7 cells (Fig. S1F). As shown in Fig. 4B–F, RBM5-AS1 overexpression significantly raised tumor incidence, whereas RBM5-AS1 knockdown restrained tumor occurrence. Also, tumors formed by injecting RBM5-AS1-overexpressing cells had a larger size and a heavier weight than the tumors of control group, while tumors generated by injecting the RBM5-AS1-knocked down cells were much smaller and lighter (Fig. 4C, G, H). Notably, only when 1 × 10^4^ cells with RBM5-AS1 overexpression were injected into nude mice, were tumors initiated (Fig. 4B, C). In the tumor sections from mice injected with equal amount of cells (1 × 10^6^), the cell proliferation, as evaluated by cell mitosis (Fig. 4I, upper) and the ratio of Ki-67-positive tumor cells (Fig. 4I, lower), was also accelerated in xenografts from RBM5-AS1-overexpressing group while attenuated in tumors of RBM5-AS1-koncked down group. Additionally, RBM5-AS1-overexpressing tumors exhibited higher expression of pluripotent transcription factors while RBM5-AS1-knocked down tumors showed lower expression compared to the control group (Fig. 4J, K). In summary, these findings indicated that RBM5-AS1 promotes stemness and tumorigenicity of breast cancer cells.

### RBM5-AS1 initiates Wnt/β-catenin signaling in breast cancer cells

**Fig. 5:**
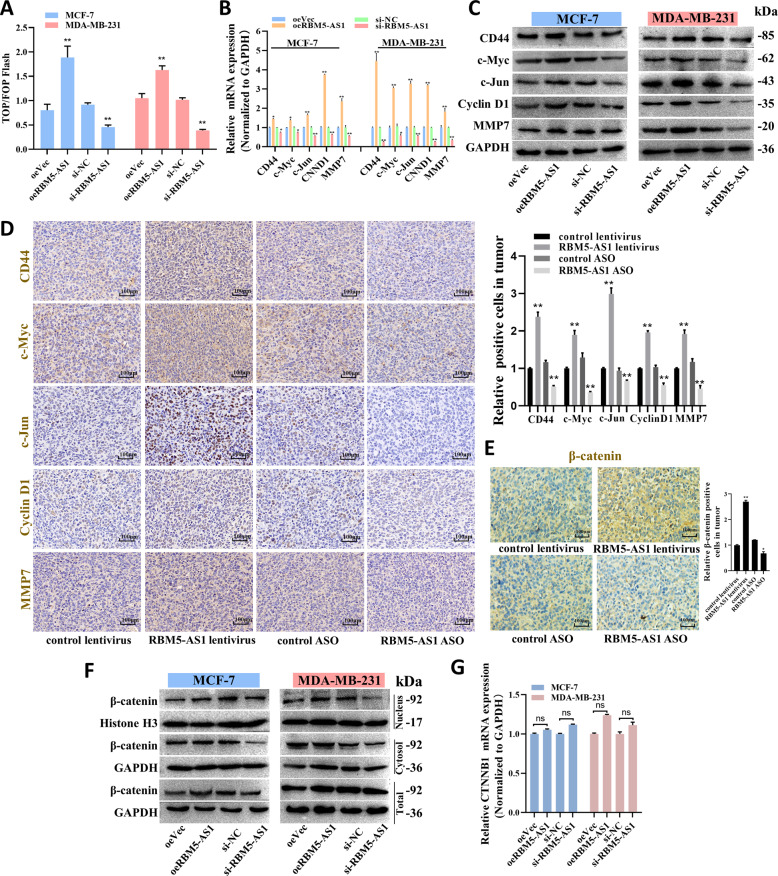
RBM5-AS1 activates Wnt/β-catenin signaling and upregulates β-catenin protein levels in breast cancer cells. **A** The activation of the WNT/β-catenin signaling was evaluated using a TOP/FOP flash assay. mRNA (**B**) and protein (**C**) levels of genes downstream of Wnt signaling in oeVec, oeRBM5-AS1, si-NC, or si-RBM5-AS1 transfected MCF-7 and MDA-MB-231 cells. **D** Representative IHC images of Wnt signaling proteins in mice tumor sections. Scale bar, 100 μm. **E** Representative IHC images of β-catenin in mice tumor sections. Scale bar, 100 μm. **F** Protein levels of β-catenin in the nuclei, cytosol, and whole cells in oeVec, oeRBM5-AS1, si-NC, or si-RBM5-AS1 transfected MCF-7 and MDA-MB-231 cells. **G** mRNA levels of β-catenin (CTNNB1) in oeVec, oeRBM5-AS1, si-NC, or si-RBM5-AS1 transfected MCF-7 and MDA-MB-231 cells. All data are shown as the mean ± SEM. *P < 0.05 and **P < 0.01 by two-tailed Student’s *t*-test.

Subsequently, we sought to identify the mechanism underlying RBM5-AS1 regulating breast cancer functions. Wnt/β-catenin pathway has been identified as a regulatory target of RBM5-AS1 in colon cancer, because RBM5-AS1 could physically interact with β-catenin to help organize the transcriptional complexes [9]. To address whether Wnt/β-catenin signaling could be activated by RBM5-AS1 in breast cancer cells, we conducted the TOP/FOP flash reporter assay to evaluate Wnt signaling activity. As shown, we detected the stronger activation of Wnt signaling in RBM5-AS1-overexpressing cells but reduced luciferase activity in RBM5-AS1-knocked down cells (Fig. 5A). In addition, upregulation of RBM5-AS1 promoted Wnt signaling pathway protein expression in both breast cancer cells and mice tumors (Fig. 5B–D). To further explore the mechanism underlying RBM5-AS1 activating Wnt/β-catenin, we started by examining whether RBM5-AS1 could alter β-catenin protein levels in the nuclei, cytosol and whole cell of MCF-7 and MDA-MB-231 cells. Indeed, we found β-catenin levels were elevated in mice tumor section from RBM5-AS1-overexpressing group (Fig. 5E), we also noticed that β-catenin levels were upregulated mainly in the nucleus and whole cells of RBM5-AS1-overexpressing cells (Fig. 5F). However, CTNNB1 (β-catenin gene) expression did not show any significant changes after RBM5-AS1 overexpression or knockdown (Fig. 5G). Therefore, these data suggested that RBM5-AS1 might exerted biological functions by activating Wnt/β-catenin signaling.

### RBM5-AS1 promotes β-catenin accumulation by inhibiting AXIN1 expression and facilitates β-catenin function through organizing β-catenin-TCF4 transcription complex

**Fig. 6:**
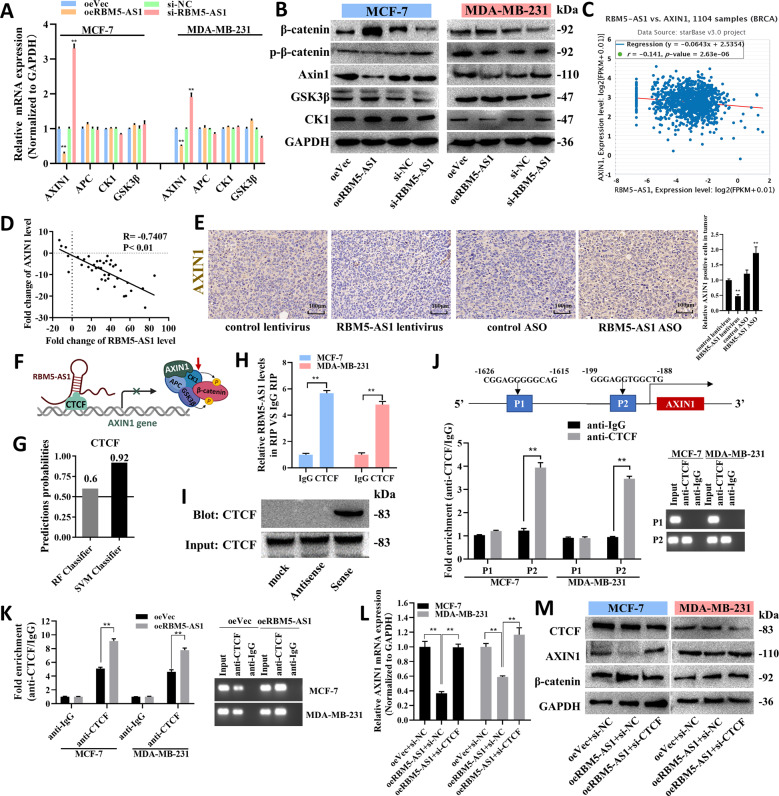
RBM5-AS1 promotes accumulation of β-catenin through inhibiting a β-catenin destruction complex component, AXIN1. mRNA (**A**) and protein (**B**) levels of β-catenin destruction complex components in oeVec, oeRBM5-AS1, si-NC, or si-RBM5-AS1 transfected MCF-7 and MDA-MB-231 cells. **C** Spearman correlation analysis of RBM5-AS1 and AXIN1 mRNA levels in 1104 human breast cancer tissues according to the TCGA project extracted from StarBase 3.0. **D** The correlation between RBM5-AS1 and AXIN1 in 40 paired breast cancer tissues was analyzed with Spearman. **E** Representative images of AXIN1 IHC staining in mice tumor sections. Scale bar, 100 μm. **F** Schematic diagram of the hypothesis that RBM5-AS1 inhibits AXIN1 expression by recruiting CTCF to the promoter region of AXIN1. **G** RPISeq software was used to predict the interaction probabilities of RBM5-AS1 and CTCF (RF and SVM > 0.5 were considered positive). **H** The interaction of RBM5-AS1 and CTCF was validated via RIP assays with CTCF antibody (IgG served as a negative control). **I** Proteins pulled down by biotin-tagged RBM5-AS1 sense or antisense were detected using CTCF antibody. **J** Putative CTCF binding sites at the AXIN1 promoter region (upper). Enrichment of CTCF on AXIN1 promoter relative to IgG in MCF-7 and MDA-MB-231 cells examined by ChIP assays (lower left) and PCR analysis (lower right). −199 to −188 bp fragment of the AXIN1 promoter contains the CTCF binding site. **K** Enrichment of CTCF on AXIN1 promoter relative to IgG in oeVec or oeRBM5-AS1 transfected MCF-7 and MDA-MB-231 cells was detected by ChIP-qPCR assays (left) and PCR analysis (right). AXIN1 mRNA levels (**L**), CTCF, AXIN-1 and β-catenin protein levels (**M**) in si-NC/oeVec or oeRBM5-AS1 or oeRBM5-AS1 plus si-CTCF transfected MCF-7 and MDA-MB-231 cells. All data are shown as the mean ± SEM. *P < 0.05 and **P < 0.01 by two-tailed Student’s *t*-test.

To reveal that how could RBM5-AS1 increase β-catenin protein levels without affecting CTNNB1 expression in breast cancer cells. We turned our attention to the regulation of β-catenin destruction complex. As presented in Fig. 6A, B, RBM5-AS1 negatively regulated the expression of AXIN1, a core component of the β-catenin destruction complex. The negative correlation between RBM5-AS1 and AXIN1 expression was identified in breast cancer tissues from Starbase database or collected by us (Fig. 6C, D) and the IHC staining in mice tumor section (Fig. 6E). It has been reported that lncRNA-LALR1 could inhibit AXIN1 expression by recruiting CTCF, a transcriptional repressor of AXIN1, to the promoter region of AXIN1. Therefore, we explored whether RBM5-AS1 repressed AXIN1 expression by a similar mechanism (Fig. 6F). RPISeq (http://pridb.gdcb.iastate.edu/RPISeq/) was used to predict the interaction propensities between RBM5-AS1 and CTCF. It showed that Random Forest (RF) and Support Vector Machine (SVM) were both higher than 0.5 (Fig. 6G). RIP-qPCR assays, RNA pull-down and Western blotting assays showed CTCF binds to RBM5-AS1 (Fig. 6H) and the CTCF protein could be pulled down by sense RBM5-AS1 (Fig. 6I). ChIP assay identified that CTCF was enriched at the promoter region within −199 to −188 bp upstream from AXIN1 transcriptional start site (Fig. 6J) and overexpression of RBM5-AS1 enhanced the enrichment of CTCF at the AXIN1 promoter region (Fig. 6K), while CTCF knockdown (Fig. S1G) prevented the decrease of AXIN1 and upregulation of β-catenin caused by RBM5-AS1 overexpressing (Fig. 6L, M).

**Fig. 7:**
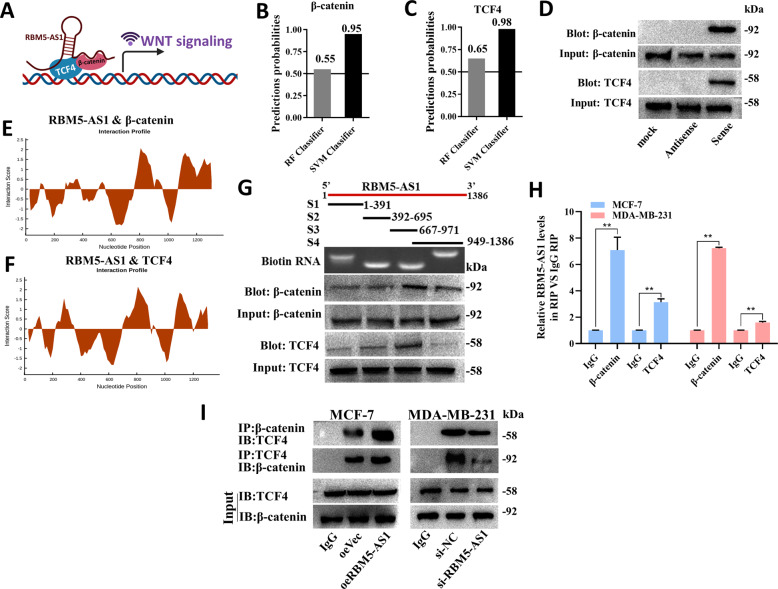
RBM5-AS1 facilitates β-catenin function through organizing β-catenin-TCF4 transcription complex. **A** Schematic diagram of the hypothesis that RBM5-AS1 promotes β-catenin/TCF4 interaction via binding with them to activate Wnt/β-catenin signaling. The possibility of interaction between RBM5-AS1 and β-catenin (**B**)/TCF4 (**C**) was predicted by RPISeq predictions. **D** Proteins pulled down by biotin-tagged RBM5-AS1 sense or antisense were detected using β-catenin/TCF4 antibody. The interaction between β-catenin (**E**)/TCF4 (**F**) protein and RBM5-AS1 was predicted using catRAPID. **G** Mapping of β-catenin/TCF4-binding domains of RBM5-AS1. Schematic of RBM5-AS1 full-length and truncated fragments (upper); agarose gel electrophoretogram of biotin-tagged RNA of truncated RBM5-AS1 (middle); western blotting of β-catenin/TCF4 in RNA-pulldown samples bound with different RBM5-AS1 fragments (lower). **H** The interaction between RBM5-AS1 and β-catenin/TCF4 was validated by RIP assays with β-catenin/TCF4 antibody (IgG served as a negative control). **I** Altered binding activity between β-catenin and TCF4 was detected in MCF-7 cells with RBM5-AS1-overexpression and MDA-MB-231 cells with RBM5-AS1-knockdown using co-IP analysis. All data are shown as the mean ± SEM. *P < 0.05 and **P < 0.01 by two-tailed Student’s *t*-test.

Moreover, we studied whether RBM5-AS1 could act as a scaffold for β-catenin-TCF4 complex (Fig. 7A). RPISeq analysis predicted the potential interaction probabilities between RBM5-AS1 and β-catenin/TCF4 (Fig. 7B, C). As shown in Fig. 7D, both β-catenin and TCF4 could be captured by biotin-labeled sense RBM5-AS1. CatRAPID fragments, showed that β-catenin and TCF4 proteins might bind to the 838-893 nt positions of the RBM5-AS1 sequence with high propensities (Fig. 7E, F). We also constructed a series of RBM5-AS1 truncated fragments to map the β-catenin/TCF4 binding region on RBM5-AS1. By RNA pulldown assay, we found that the 667 to 971 nt fragment was responsible for RBM5-AS1 interaction with β-catenin/TCF4 (Fig. 7G). RIP assays further confirmed the interaction between RBM5-AS1 and β-catenin/TCF4 (Fig. 7H). Co-IP assays demonstrated that RBM5-AS1 overexpression significantly enhanced the β-catenin/TCF4 interactions in MCF-7 cells, while RBM5-AS1 knockdown generated the contrary effects in MDA-MB-231 cells (Fig. 7I). Altogether, these data revealed that RBM5-AS1 not only blocked the degradation of β-catenin, but also facilitated the binding between β-catenin and TCF4, which subsequently triggered Wnt/β-catenin pathway.

### RBM5-AS1 facilitates cell proliferation and stemness through regulating β-catenin to activate Wnt signaling in breast cancer

**Fig. 8:**
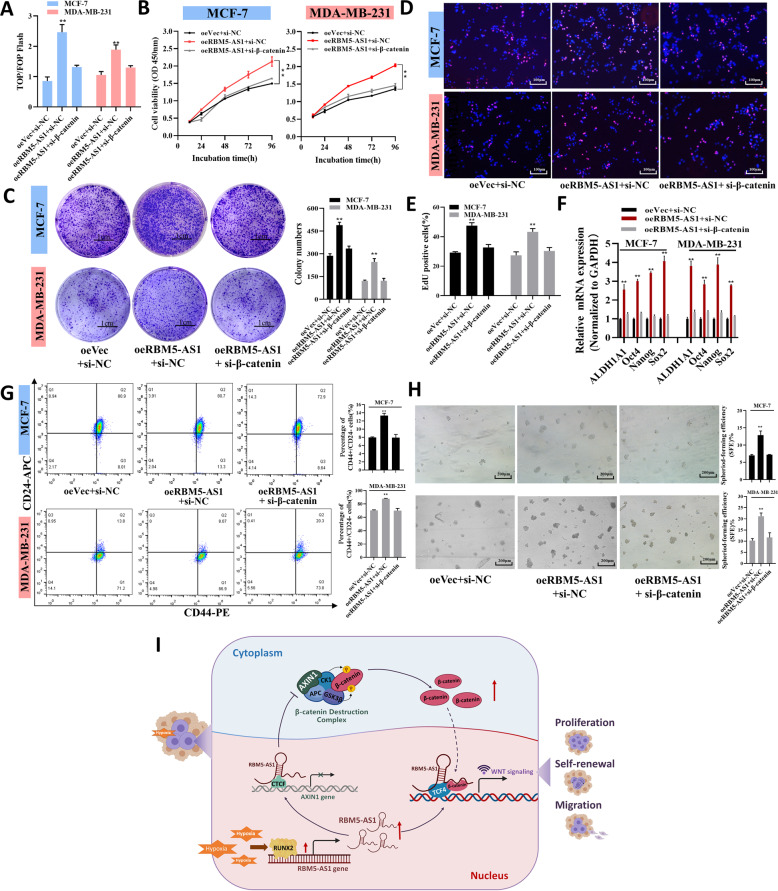
Knockdown of β-catenin restores the proliferative and pro-stemness functions of RBM5-AS1 in breast cancer cells. **A** The activation of the WNT/β-catenin signaling was detected using a TOP/FOP flash assay. Proliferative abilities of oeVec plus si-NC, oeRBM5-AS1 plus si-NC, or oeRBM5-AS1 plus si-β-catenin transfected MCF-7 and MDA-MB-231 cells were evaluated by CCK-8 assays (**B**), plate colony assays (**C**, Scale bar, 1 cm), and EdU assays (**D** and **E**, Scale bar, 100 μm). **F** mRNA levels of pluripotent transcription factors in oeVec plus si-NC, oeRBM5-AS1 plus si-NC, or oeRBM5-AS1 plus si-β-catenin transfected MCF-7 and MDA-MB-231 cells. **G** Proportion of CD44+CD24− cells in oeVec plus si-NC, oeRBM5-AS1 plus si-NC, or oeRBM5-AS1 plus si-β-catenin transfected MCF-7 and MDA-MB-231 cells. **H** Sphere-formation abilities of oeVec plus si-NC, oeRBM5-AS1 plus si-NC, or oeRBM5-AS1 plus si-β-catenin transfected MCF-7 and MDA-MB-231 cells. Scale bar, 200 μm. **I** During breast tumorigenesis, RBM5-AS1 was upregulated by a hypoxia-induced transcriptional factor, RUNX2. RBM5-AS1 promoted proliferation, stemness, migration, and invasion of breast cancer cells via activating the WNT/β-catenin pathway in two ways. On the one hand, RBM5-AS1 could prevent β-catenin degradation by repressing transcription of AXIN1, a core component of the β-catenin destruction complex, which in turn facilitated β-catenin nucleus accumulation. On the other hand, RBM5-AS1 could function as a scaffold for β-catenin-TCF4 complex, leading to transcriptional activation of WNT/β-catenin signaling. All data are shown as the mean ± SEM. *P < 0.05 and **P < 0.01 by two-tailed Student’s *t*-test.

Finally, we intended to validate the contribution of RBM5-AS1 to proliferation and stemness of breast cancer cells was mediated by regulating β-catenin to stimulate Wnt/β-catenin signaling, rescue experiments were proceeded (Fig. S1H). By using TOP/FOP-Flash assay, we found the enhanced Wnt activity by overexpressing RBM5-AS1 was attenuated by β-catenin knockdown (Fig. 8A). Also, we observed the proliferative effect of RBM5-AS1 was reversed by silencing β-catenin (Fig. 8B–E). Moreover, knockdown of β-catenin abolished the increased pluripotent transcription factors expression, CD44+CD24− cells ratio, and sphere-formation efficiency by RBM5-AS1 overexpression (Fig. 8F–H). To sum up, RBM5-AS1 enhances the proliferation and stemness of breast cancer cell via β-catenin.

## Discussion

At present, the overall therapeutic effect for breast cancer remains unsatisfactory. In order to uncover new therapeutic methods, it is of great importance to study the mechanism of breast cancer development and progression based on tumor molecular biology. In fact, increasing studies have been focusing on the functions and regulations of lncRNAs to discover new targets for the diagnosis and treatment of cancers [16].

RBM5-AS1 is a nuclear-retained lncRNA [9] that is located on 3p21.31with a total length of 1386 bp. However, there has been a paucity of literature about RBM5-AS1 regarding its functions up to now. In different cancers, RBM5-AS1 was found to promote stem-like properties of colon cancer cells by recruiting β-catenin to the promoter regions of Wnt signaling target genes [9], RBM5-AS1 facilitates osteosarcoma cells [11], oral squamous cell carcinoma cells [12], and hepatocarcinoma cells [10] proliferation and metastasis through regulating RBM5, miR-1285-3p/YAP1 axis and silencing miR-132/212, respectively. RBM5-AS1 has also been demonstrated to inhibit bone cell apoptosis and promoted fracture healing by upregulating of β-catenin [17]. Previously, we detected that RBM5-AS1 was significantly increased in BCSCs and poorly differentiated breast cancer tissues compared with non-BCSCs and paracancerous normal tissues, respectively [6]. However, the expression and function of RBM5-AS1 in breast cancer, and their underlying regulatory mechanisms were still unknown. Thus, we first verified the upregulation of RBM5-AS1 in different breast cancer cell lines, BCSCs, and breast cancer tissues. Besides, we found positive correlations between RBM5-AS1 level and tumor grade, tumor histological, and tumor size. Gain- and loss-of-function experiments were performed next and showed that RBM5-AS1 is crucial for the proliferative, stemness maintenance, migratory and invasive capacities of breast cancer cells in vitro, and tumorigenicity of breast cancer cells in vivo. Collectively, the current work reveals the mechanisms and expands our understanding on the roles of RBM5-AS1 in cancer progression.

Overactivation of the Wnt/β-catenin signaling pathway is recognized as cancer hallmarks and critical processes during tumor development. Consistent with the previous findings [9, 17], we demonstrated that RBM5-AS1 could upregulate and interact with β-catenin, thus initiates Wnt/β-catenin signaling. Moreover, we provided insight into the mechanism by which RBM5-AS1 increases the levels of β-catenin and facilitates β-catenin organizing transcriptional complex. On the one hand, we revealed that RBM5-AS1 inhibited the formation of β-catenin destruction complex via downregulating the expression of its component, AXIN1, which caused the β-catenin accumulation in the cytoplasm and its subsequent nucleus translocation. On the other hand, we found the specific binding sites between RBM5-AS1 and β-catenin/TCF4 and demonstrated that RBM5-AS1 could reinforce TCF4-β-catenin interaction as a scaffold. Our findings suggested that RBM5-AS1/β-catenin axis may have crucial and broad functions in the occurrence and progression of various cancers.

Our study also provided the first evidence that RUNX2 is a transcriptional factor of RBM5-AS1 and mediated the hypoxia-induced RBM5-AS1 upregulation. Hypoxic tumor microenvironment, resulted from insufficient vascularization and high tumor metabolic and proliferative rates, facilitates the progression and malignant transformation of multiple solid tumors including breast cancer [8]. Notably, a specific group of lncRNAs named hypoxia-responsive lncRNAs (HRLs) can be modulated by hypoxic tumor microenvironment, such as NORAD, RAB11B-AS1, and AC020978, are involved in cancer progression [18–20]. Nevertheless, the roles of HRLs in breast cancer remain undefined at present. In the current study, we pointed out to RBM5-AS1 as a novel player taking part in the response of breast cancer cells to hypoxia. Different from hypoxia-inducible factor (HIF)-dependent HRLs, we demonstrated that RBM5-AS1 was transcribed by RUNX2 instead of HIF under hypoxic condition. RUNX2 is a transcription factor implicated in tumor growth, invasion, and metastasis [21]. Previous studies have shown that RUNX2 could be upregulated by hypoxia in prostate cancer [21]. Besides, several studies have reported that Wnt/β-catenin signaling could be activated by hypoxia in cancer [22–24], but the mechanism has not well been documented, especially in breast cancer. Our data implied that RBM5-AS1 might mediate the hypoxia-induced activation of Wnt/β-catenin signaling and consequently, facilitated cell proliferation, self-renewal, migration, and invasion during breast cancer development. The major findings of this study have been summarized in a schematic diagram (Fig. 8I).

In conclusion, hypoxia-induced RBM5-AS1 exerts a key role in the proliferation, stemness maintenance, migration, and invasion of breast cancer by promoting β-catenin accumulation and reinforcing TCF4-β-catenin interaction to trigger Wnt/β-catenin pathway. Our present work provides new evidence that RBM5-AS1 maybe used as novel biomarker for breast cancer diagnosis and blocking the RBM5-AS1/Wnt/β-catenin axis can serve as a potential therapeutic target for breast cancer therapy.

## Supplementary information

Supplemental Figure and Table legends

Figure S1

Figure S2

Supplementary Tables

Supplementary data

Reproducibility checklist

## Data Availability

The data used to support the findings of this study are available from the corresponding author upon request.
