# Supplementary material for: Hypoxia-induced lncRNA RBM5-AS1 promotes tumorigenesis via activating Wnt/β-catenin signaling in breast cancer
Source: Cell Death Dis. 2022 Feb 2;13(2):95. doi: 10.1038/s41419-022-04536-y (PMC8810931; doi:10.1038/s41419-022-04536-y)
Supplement: Supplementary file 1 — Supplemental Figure and Table legends [file 41419_2022_4536_MOESM1_ESM.docx]

**Supplementary Figure and Table legends**

**Fig. S1 Verification of overexpression and knockdown efficiencies of RUNX2, CTCF and RBM5-AS1. (A)** RUNX2 levels detected in MCF-7 and MDA-MB-231 cells transfected with oeVec, oeRUNX2, si-NC and si-RUNX2. **(B)** RBM5-AS1 levels detected in MCF-7 and MDA-MB-231 cells transfected with oeVec, oeRBM5-AS1, si-NC and si-RBM5-AS1. **(C** and **D)** RBM5 (C) and RBM6 (D) mRNA levels detected in MCF-7 and MDA-MB-231 cells transfected with oeVec, oeRBM5-AS1, si-NC and si-RBM5-AS1. **(E)** RBM5 and RBM6 protein levels detected in MCF-7 and MDA-MB-231 cells transfected with oeVec, oeRBM5-AS1, si-NC and si-RBM5-AS1. **(F)** RBM5-AS1 levels detected in MCF-7 cells transfected with control lentivirus, RBM5-AS1 lentivirus, control ASO and RBM5-AS1 ASO. **(G)** CTCF levels detected in MCF-7 and MDA-MB-231 cells transfected with si-NC and si-CTCF. **(H)** β-catenin mRNA levels detected in MCF-7 cells transfected with oeVec plus si-NC, oeRBM5-AS1 plus si-NC, or oeRBM5-AS1 plus si-β-catenin. All data are shown as the mean±SEM. **P* <0.05 and ** *P*<0.01 by two-tailed Student’s *t*-test.

**Fig. S2 RBM5-AS1 promotes breast cancer cell migration, invasion and EMT *in vitro*. (A-C)** Migration of RBM5-AS1-overexpressing or -knocked down MCF-7 (A) and MDA-MB-231 cells (B) detected by wound healing assays. Scale bar, 200 μm. Percent of wound compared to the starting point (0 hour) was analyzed from three independent experiments (C). **(D-F)** Migration and invasion of RBM5-AS1-overexpressing or -knocked down MCF-7 (D) and MDA-MB-231 cells (E) detected by transwell assays. Scale bar, 100 μm. Numbers of migrated or invaded cells were analyzed (F). **(G)** The protein levels of EMT target genes in RBM5-AS1-overexpressing or -knocked down MCF-7 and MDA-MB-231 cells. All data are shown as the mean±SEM. **P* <0.05 and ** *P*<0.01 by two-tailed Student’s *t*-test.

**Table S1. RMB5-AS1 expression and clinicopathological features in 40 patients with breast cancer**

**Table S2. Sequences of primers used for qRT-PCR, plasmid construction and ChIP-qPCR**

**Table S3. Antibodies used for western blotting (WB), immunoprecipitation (IP), and flow cytometry (FC).**
