# Supplementary figures and images for: Hypoxia-induced lncRNA RBM5-AS1 promotes tumorigenesis via activating Wnt/β-catenin signaling in breast cancer
Source: Cell Death Dis. 2022 Feb 2;13(2):95. doi: 10.1038/s41419-022-04536-y (PMC8810931; doi:10.1038/s41419-022-04536-y)

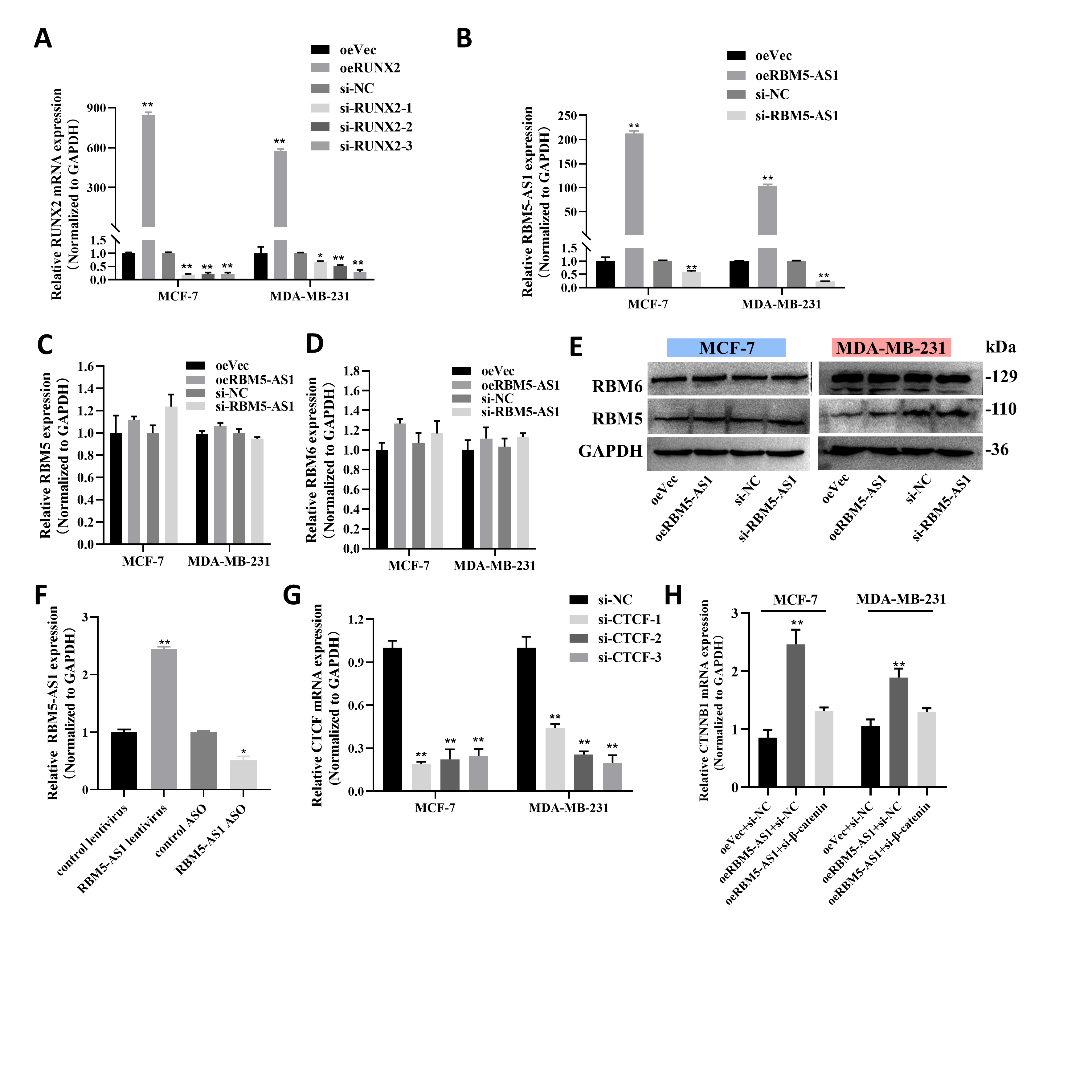

Supplement: Supplementary file 2 — Figure S1 [file 41419_2022_4536_MOESM2_ESM.tif]

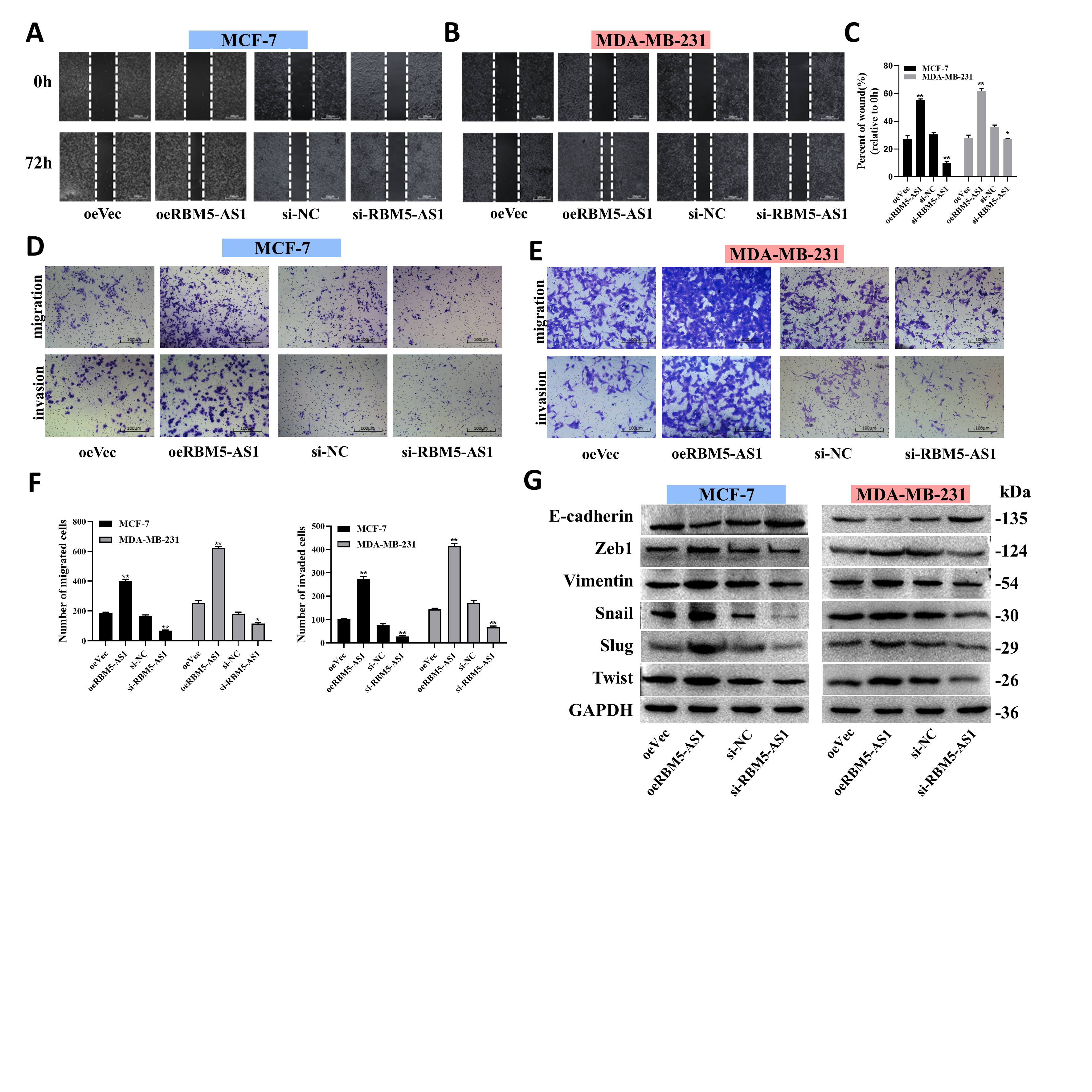

Supplement: Supplementary file 3 — Figure S2 [file 41419_2022_4536_MOESM3_ESM.tif]

Fig 2J

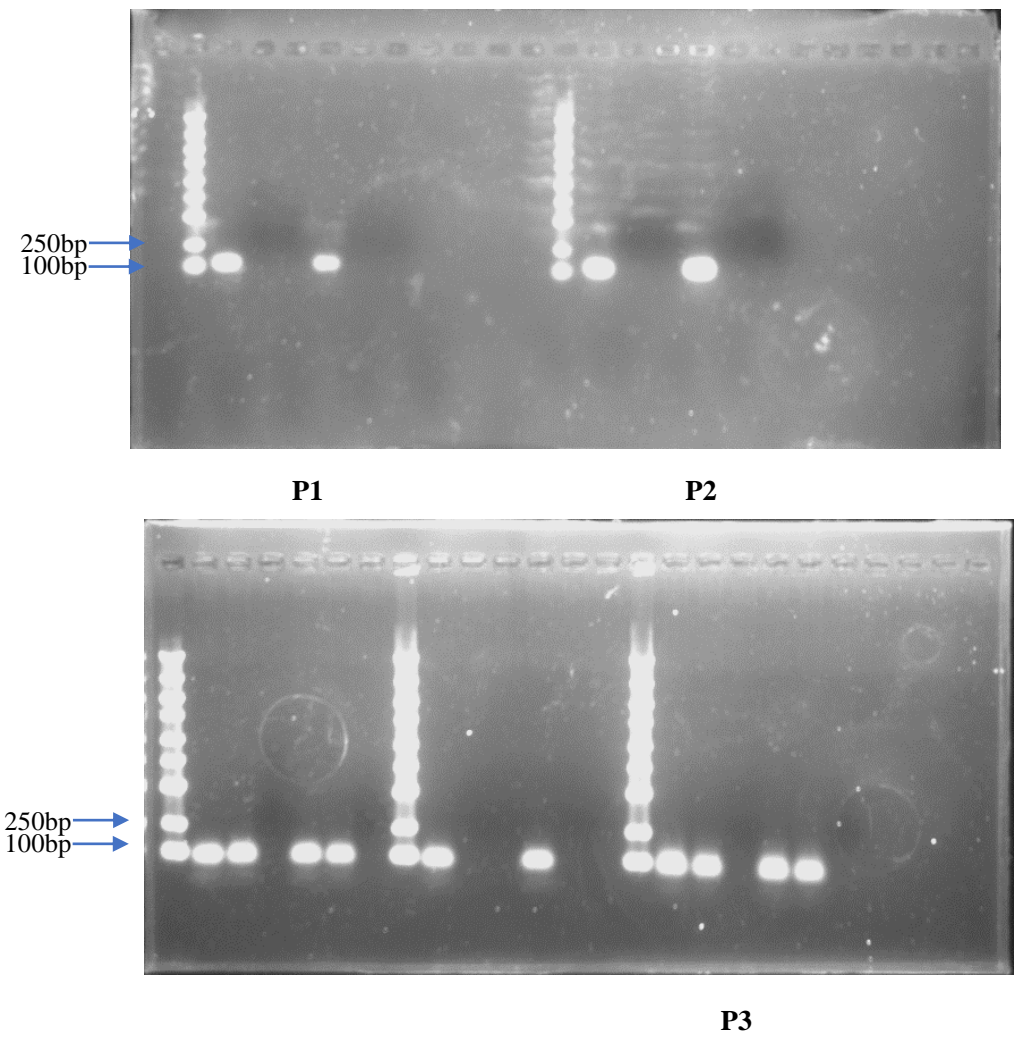

Fig 2L

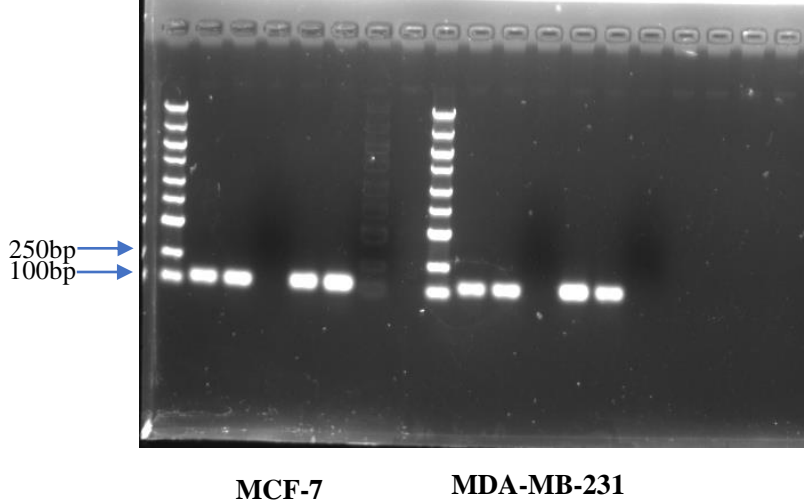

Fig 3G

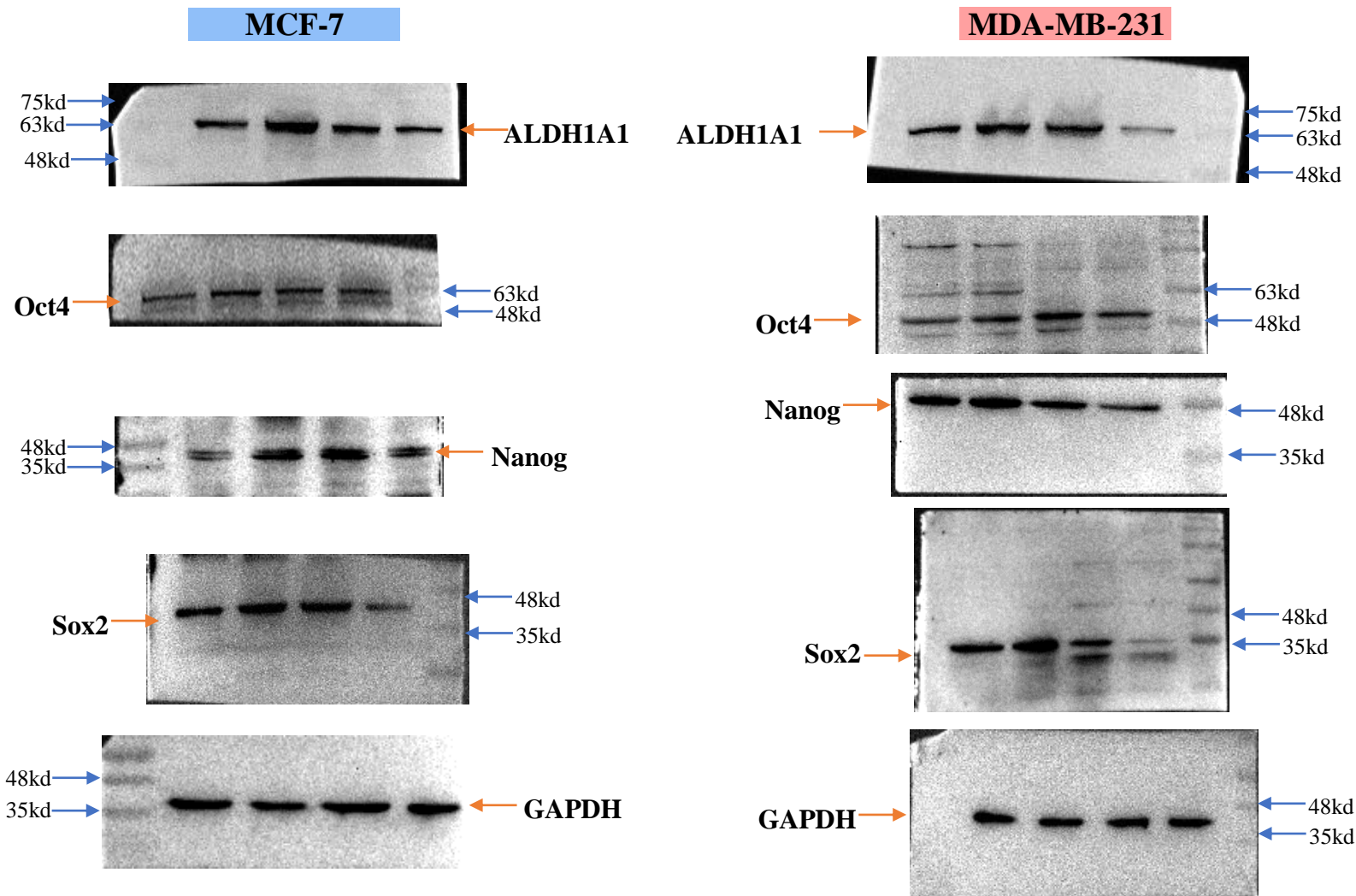

Fig 5C

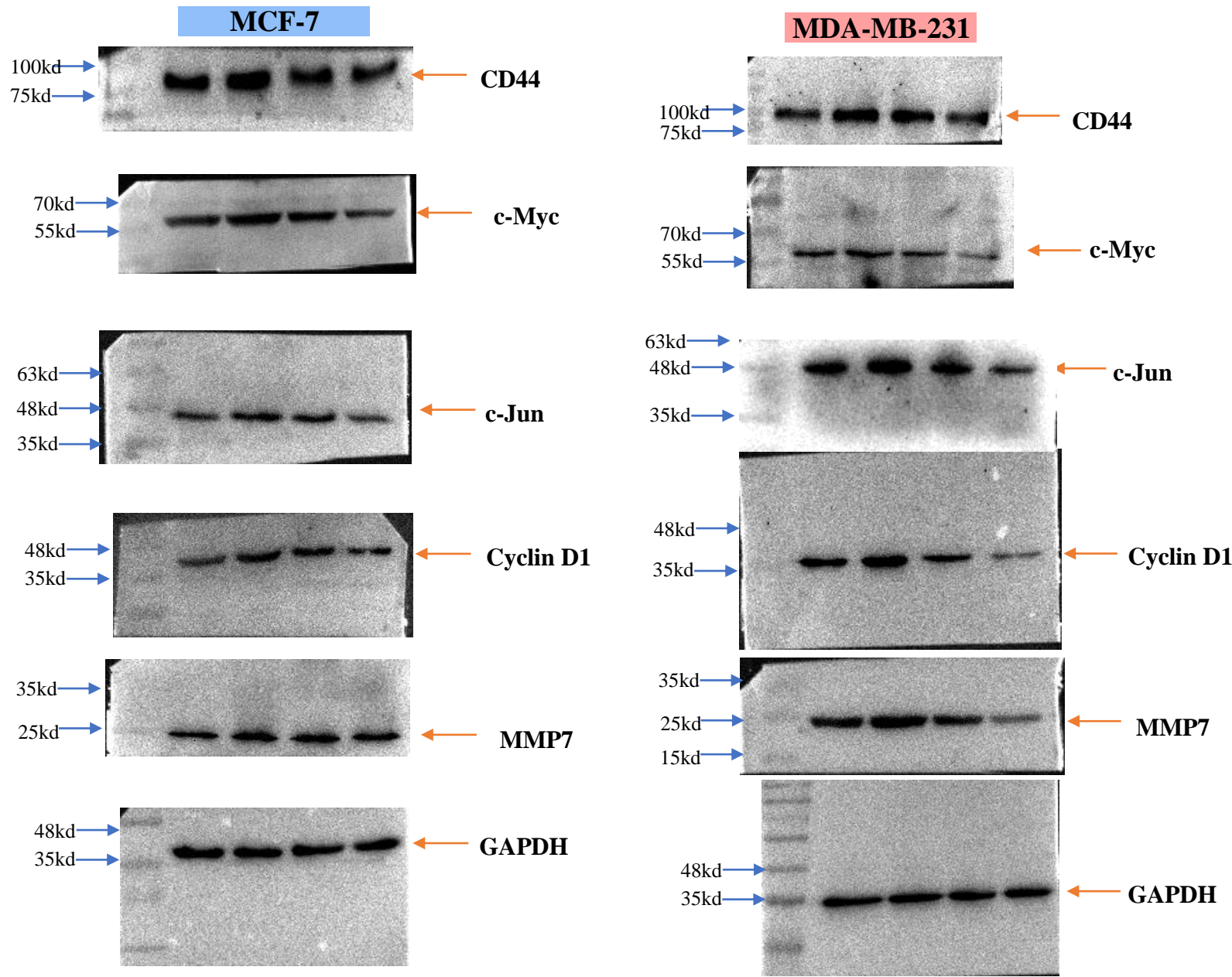

Fig 5F

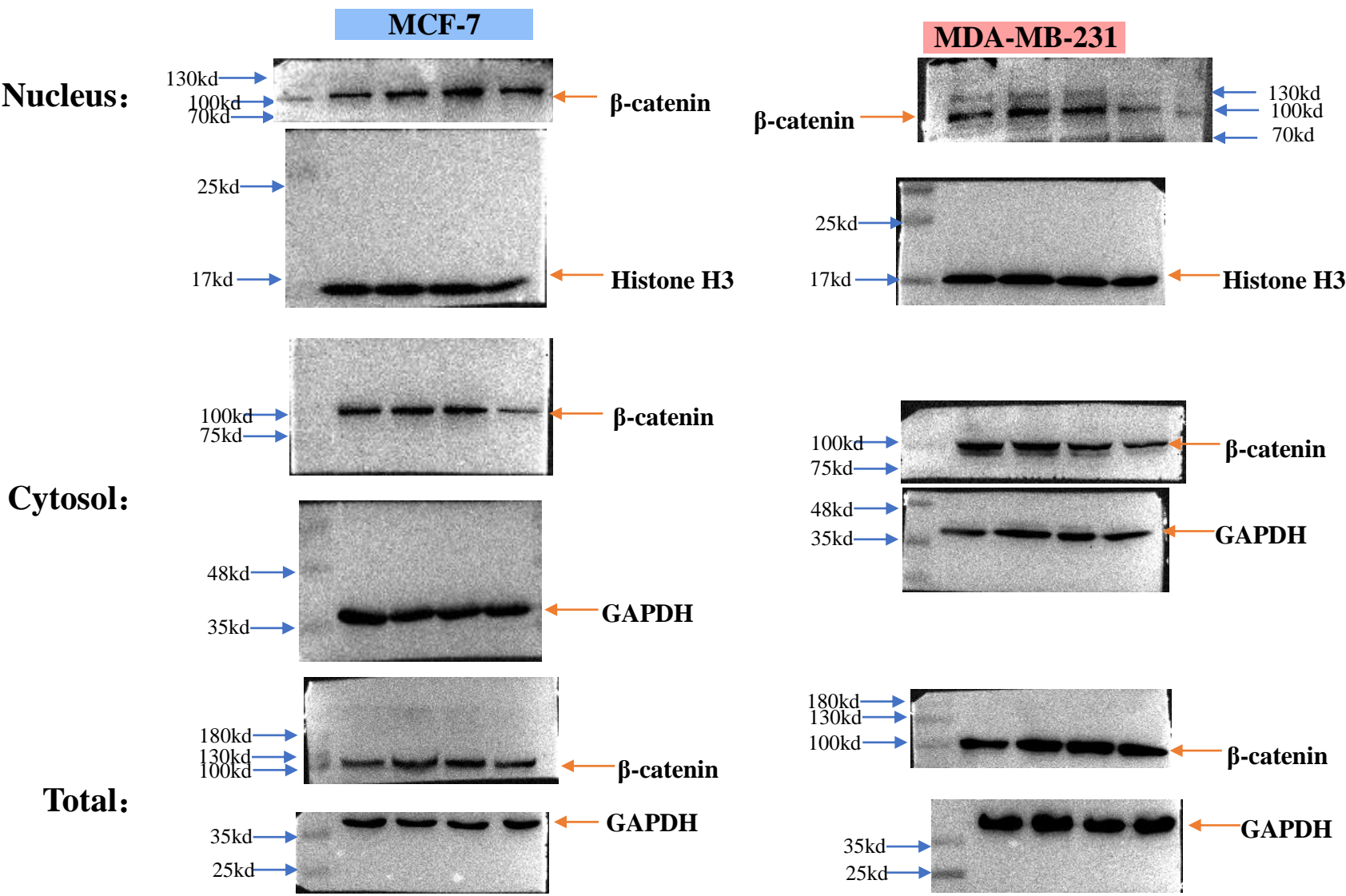

Fig 6B

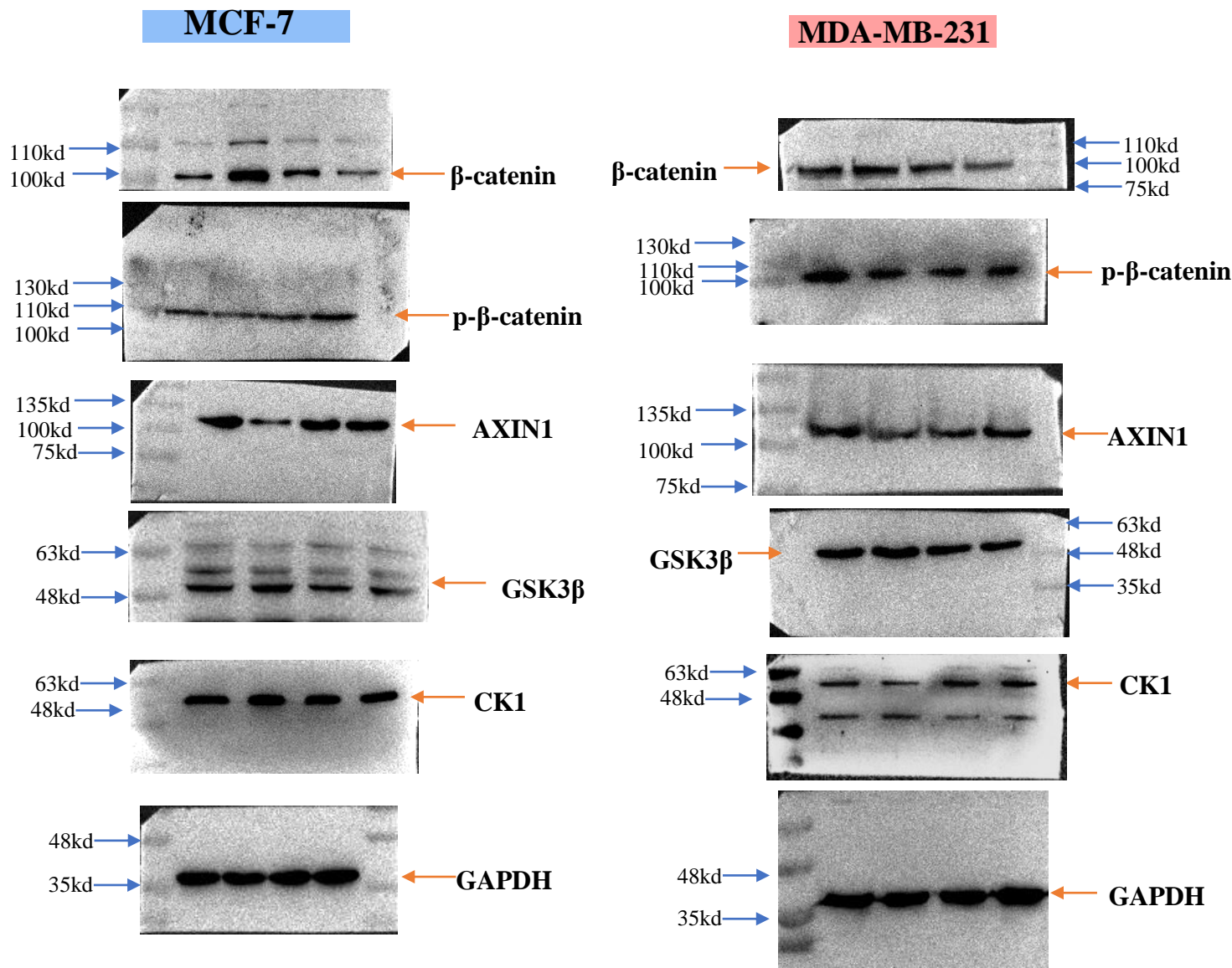

Fig 6I

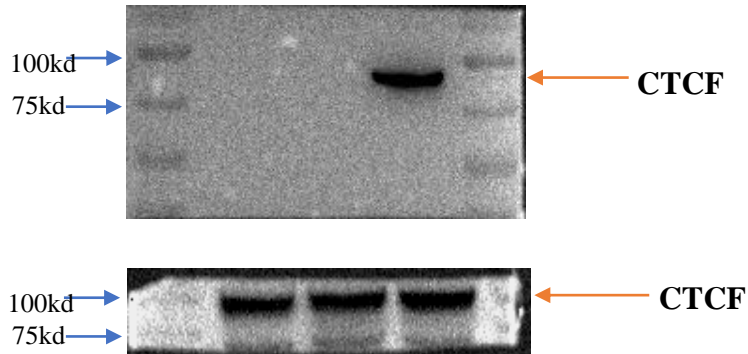

Fig 6J

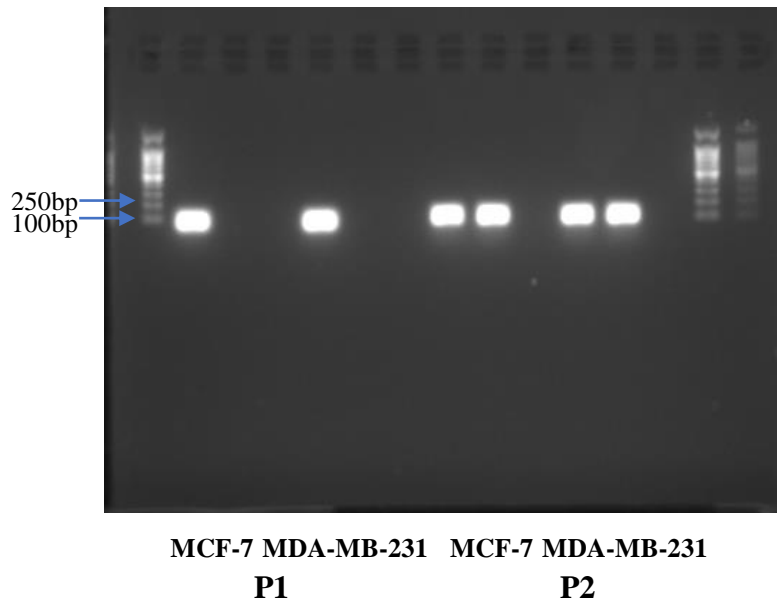

Fig 6K

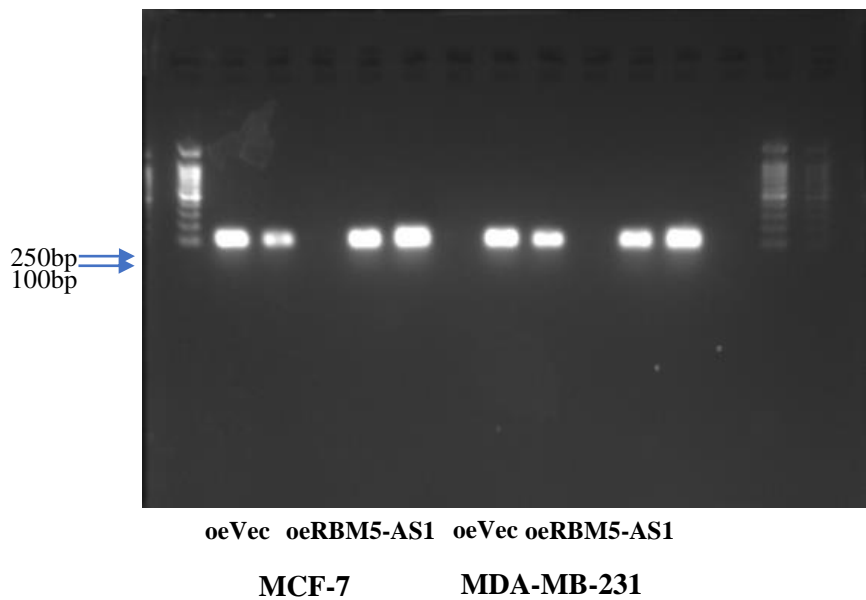

Fig 6M

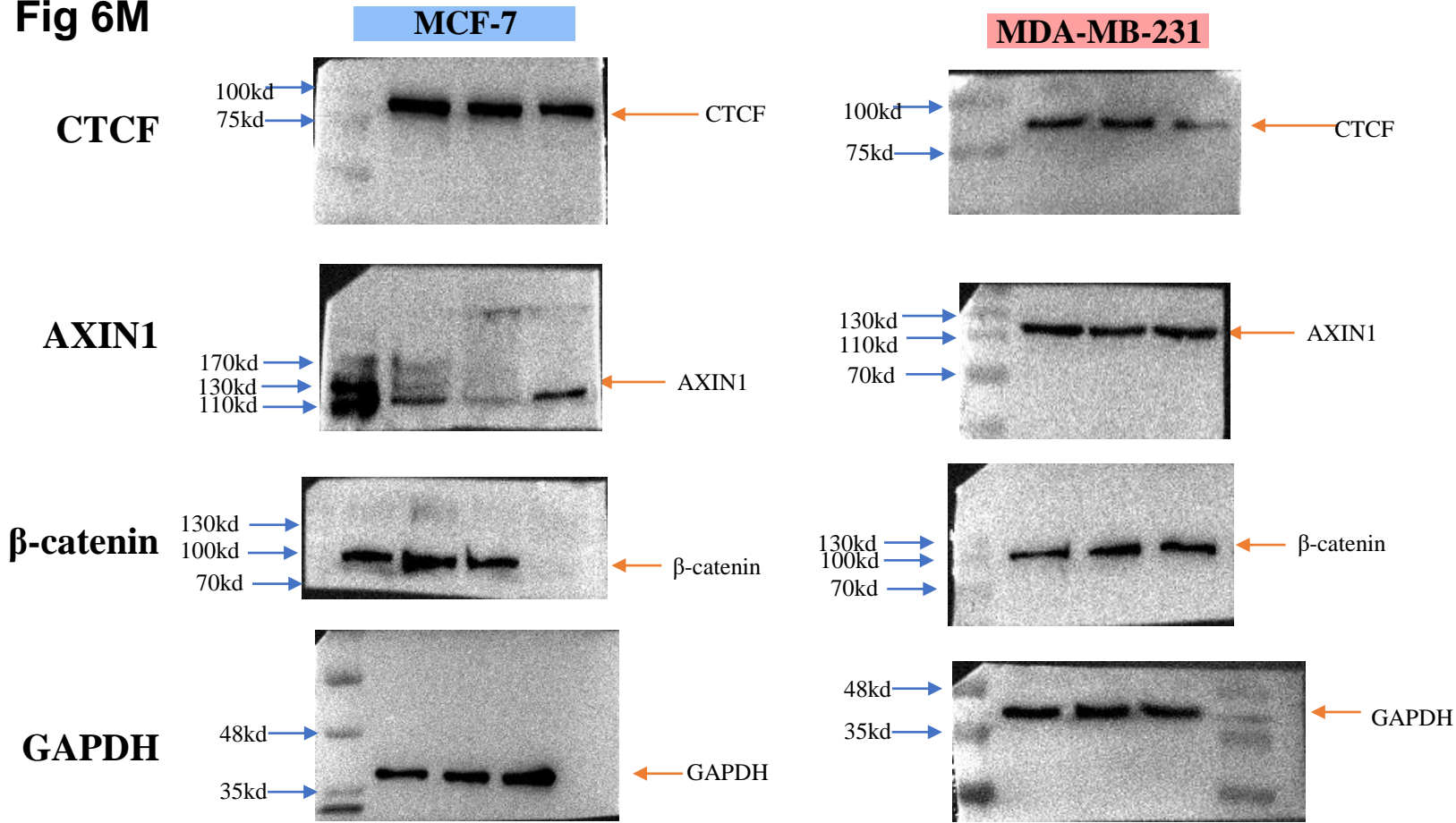

Fig 7D

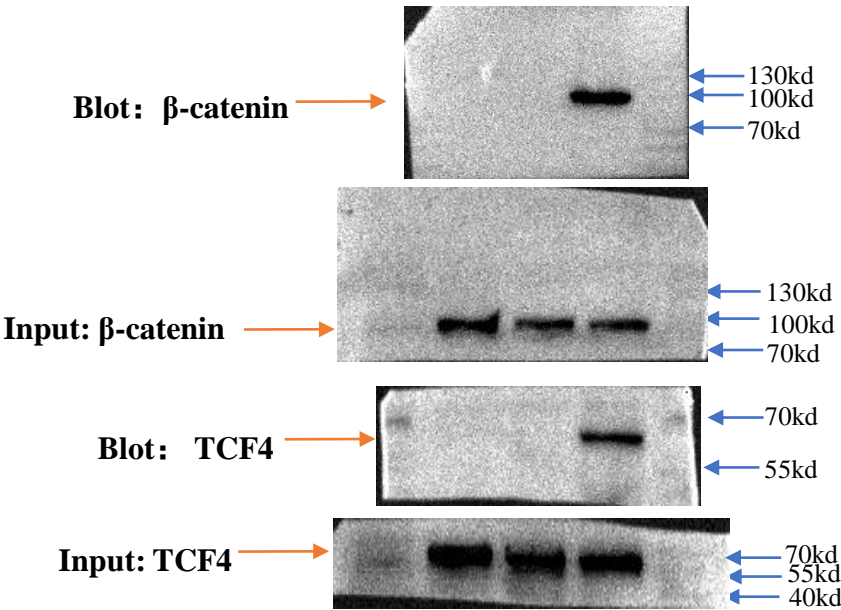

Fig 7G

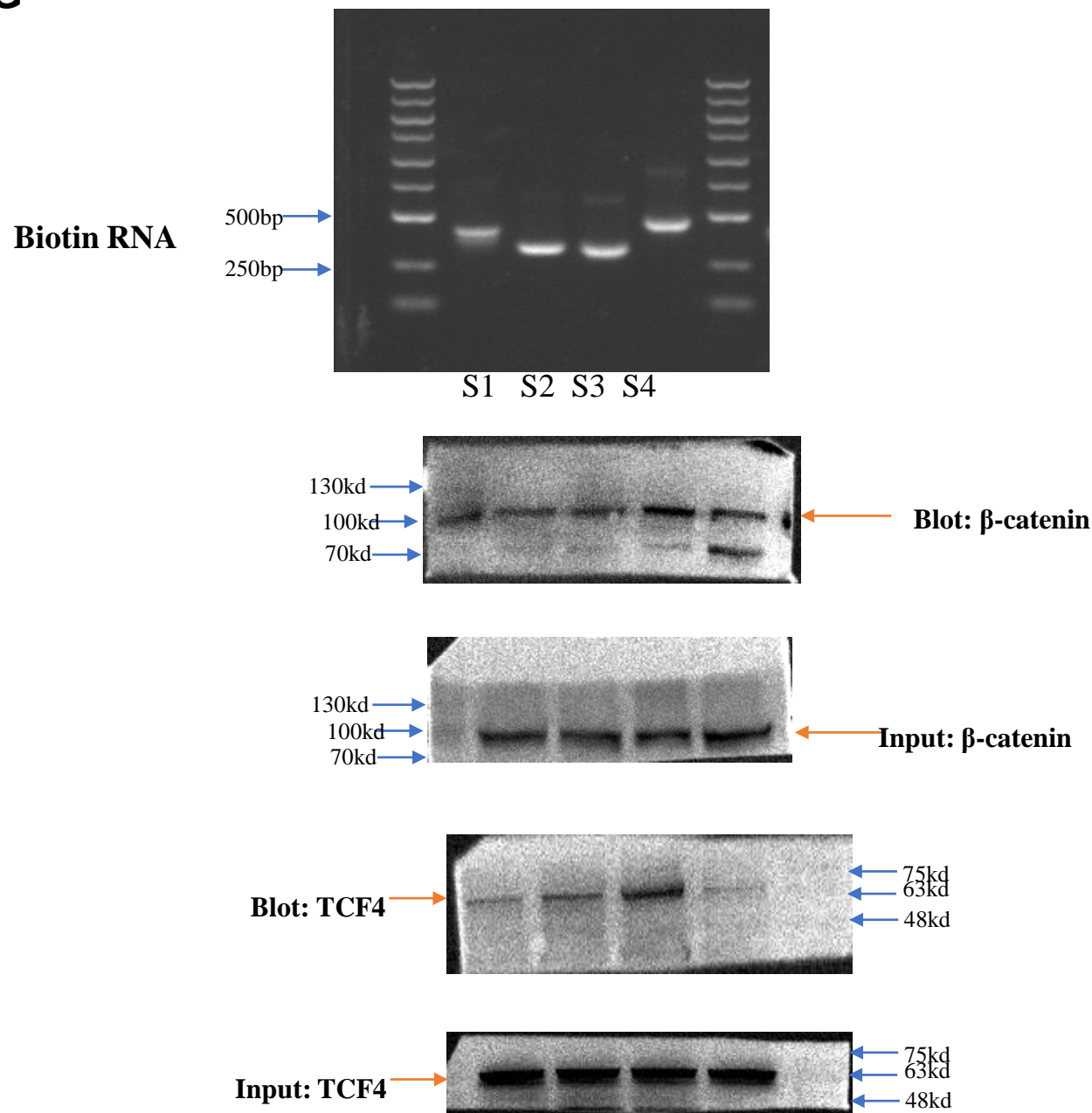

Fig 7I

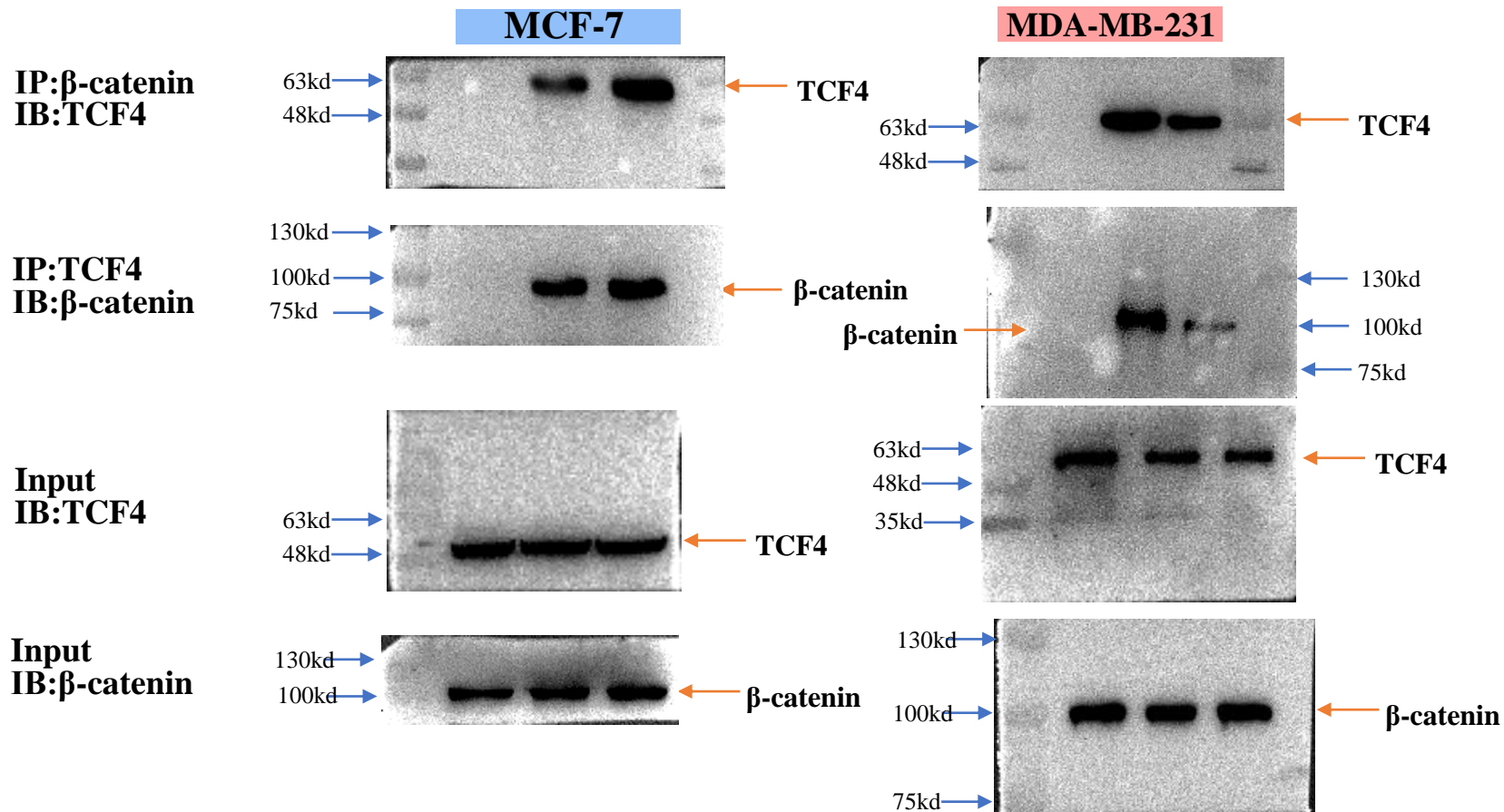

Fig S1E

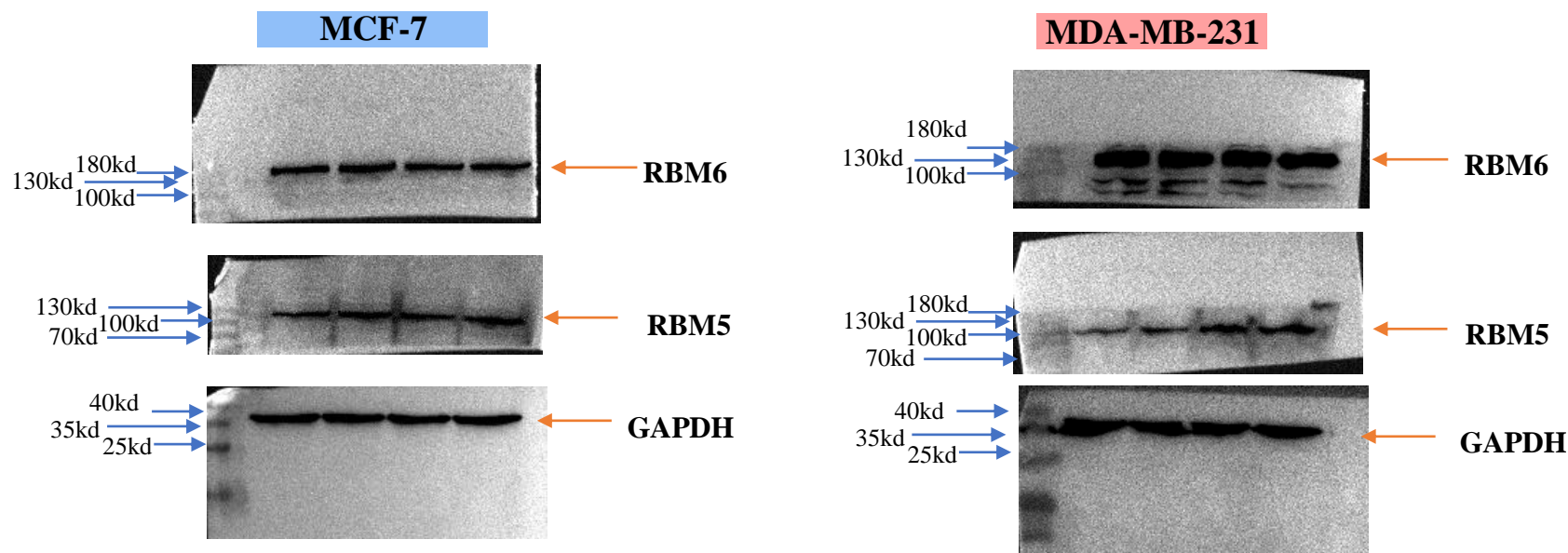

Fig S2G

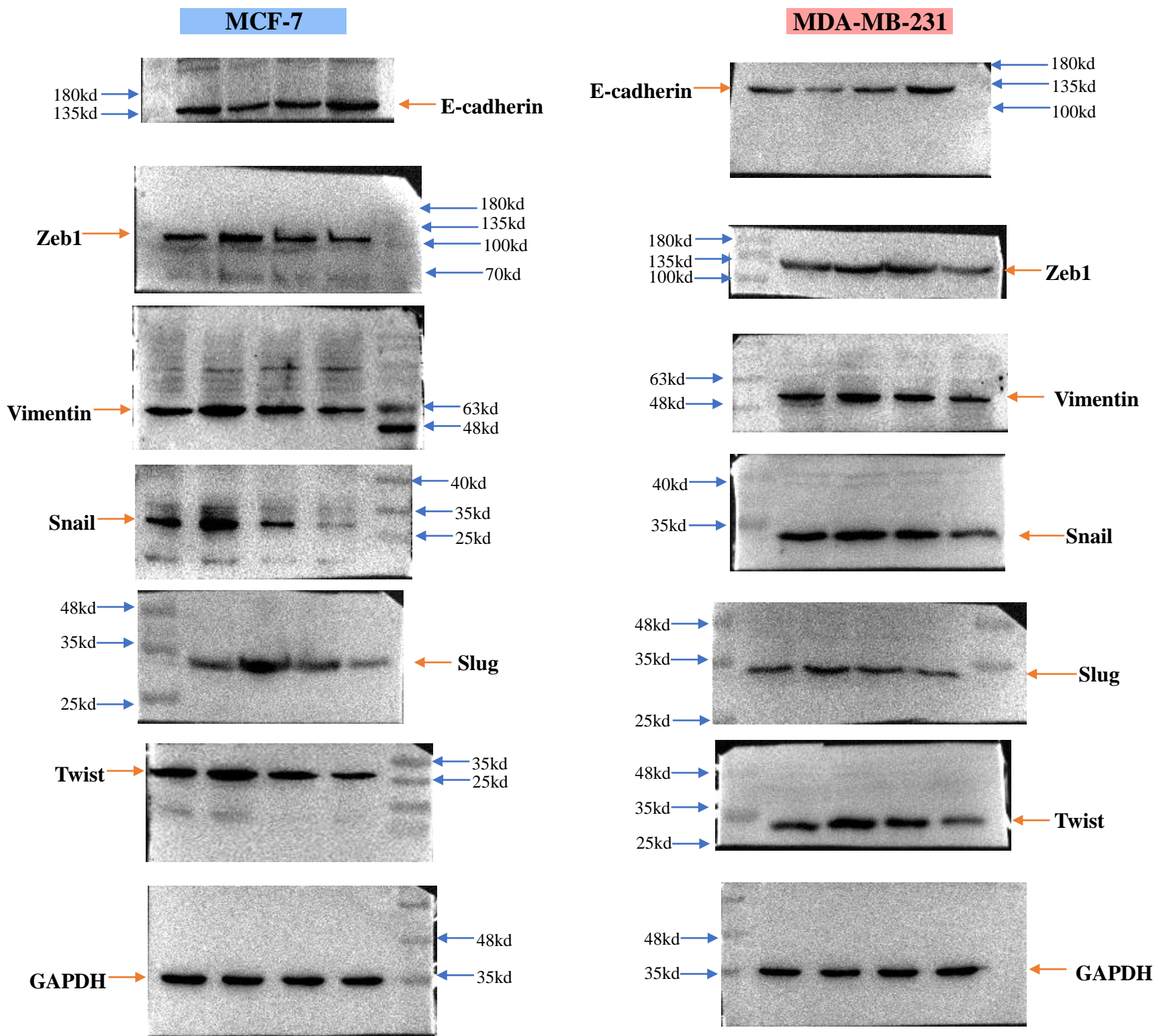

Supplement: Supplementary file 5 — Supplementary data [file 41419_2022_4536_MOESM5_ESM.pdf]
