# Supplementary material for: Hypoxia-induced lncRNA RBM5-AS1 promotes tumorigenesis via activating Wnt/β-catenin signaling in breast cancer
Source: Cell Death Dis. 2022 Feb 2;13(2):95. doi: 10.1038/s41419-022-04536-y (PMC8810931; doi:10.1038/s41419-022-04536-y)
Supplement: Supplementary file 4 — Supplementary Tables [file 41419_2022_4536_MOESM4_ESM.docx]

| **Supplementary Table S1. RMB5-AS1 expression and clinicopathological features in 40 patients with breast cancer** | | | |
| --- | --- | --- | --- |
| **Characteristics** | **Expression of RBM5-AS1** | | **p value*** |
|  | **low** | **high** |  |
| **Sex** |  |  |  |
| male | 0 | 0 |  |
| female | 20 | 20 |  |
| **Age** |  |  | 0.278 |
| ≤60 | 11 | 8 |  |
| >60 | 9 | 13 |  |
| **Grade** |  |  | 0.000** |
| I /I-II, well-differentiated | 12 | 0 |  |
| II /II-III, moderately differentiated | 8 | 10 |  |
| III, poorly differentiated | 0 | 10 |  |
| **Tumor histological** |  |  | 0.000** |
| Ductal carcinoma in situ | 16 | 3 |  |
| Invasive ductal carcinoma | 4 | 17 |  |
| **Lymph node metastasis** |  |  | 0.110 |
| Negative | 11 | 6 |  |
| Positive | 9 | 14 |  |
| **ER status** |  |  | 0.342 |
| Negative | 12 | 9 |  |
| Positive | 8 | 11 |  |
| **PR status** |  |  | 0.736 |
| Negative | 14 | 13 |  |
| Positive | 6 | 7 |  |
| **HER2 status** |  |  | 0.102 |
| Negative | 5 | 10 |  |
| Positive | 15 | 10 |  |
| **Tumor size(cm^3^)** |  |  | 0.027* |
| ≤ 6 | 14 | 7 |  |
| > 6 | 6 | 13 |  |
| Median expression level was used as a cutoff to divide the 80 patients into RBM5-AS1 low group (n = 20) and RBM5-AS1 high group (n = 20). | | | |
| Two-sided χ2 test. **P*<0.05, ** *P*<0.01. |  |  |  |

| **Supplementary Table S2. Sequences of primers used for qRT-PCR, plasmid construction and ChIP-qPCR** | |
| --- | --- |
| **Primer names** | **Sequences** |
| **Sequences of primers used for qRT-PCR** |  |
| LncSPRY4-IT1 forward | 5'-CCAGCAGTGGAACTCTGATTTG-3' |
| LncSPRY4-IT1 reverse | 5'-GCTCCACTGGGCATATTCTAAA-3' |
| LncRPPH1 forward | 5'-GAGGTCAGACTGGGCAGGAGAT-3' |
| LncRPPH1 reverse | 5'-CCTCACCTCAGCCATTGAACTC-3' |
| LncNEAT1 forward | 5'-GGGTGGTCTGAGGAGTGATGT-3' |
| LncNEAT1 reverse | 5'-CCTGGAAAATAAAGCGTTGGT-3' |
| LncOSBP2-2 forward | 5'-CATCTCACAAGGCTGCACCAG-3' |
| LncOSBP2-2 reverse | 5'-GTTTTCACTCAAAGGGCTTCATG-3' |
| LncRMRP forward | 5'-CCACTCCAAAGTCCGCCAAG-3' |
| LncRMRP reverse | 5'-GCACTGCCTGCGTAACTAGAG-3' |
| LncRBM5-AS1 forward | 5'-TTCCTCTTCATCAGCCTCACATC-3' |
| LncRBM5-AS1 reverse | 5'-GTAAAACCCTGTGCAGATTCGAG-3' |
| LncARHGDIB forward | 5'-GACCACTCTGCTGTTGATCC-3' |
| LncARHGDIB reverse | 5'-CTTGCTTATGCTGCCTCTTCAA-3' |
| LncHEIH forward | 5'-TCACATACCAGTGGCCAGAAGT-3' |
| LncHEIH reverse | 5'-GCCATTGTCTTGTTGACTCTGTT-3' |
| LncFTX forward | 5'-TTGCCTCCCTCTTTTCCGA-3' |
| LncFTX reverse | 5'-CCCACTCTCAGCACCTCATT-3' |
| GAPDH forward | 5'-GAGTCAACGGATTTGGTCGT-3' |
| GAPDH reverse | 5'-TTGATTTTGGAGGGATCTCG-3' |
| U6 forward | 5'-ATTGGAACGATACAGAGAAGATT-3' |
| U6 reverse | 5'-GGAACGCTTCACGAATTTG-3' |
| RUNX2 forward | 5'-CCGGAATGCCTCTGCTGTTATGA-3' |
| RUNX2 reverse | 5'-ACTGAGGCGGTCAGAGAACAAACT-3' |
| ALDH1A1 forward | 5'-CTGCTGGCGACAATGGAGT-3' |
| ALDH1A1 reverse | 5'-CGCAATGTTTTGATGCAGCCT-3' |
| OCT4 forward | 5'-GGGAGATTGATAACTGGTGTGTT-3' |
| OCT4 reverse | 5'-GTGTATATCCCAGGGTGATCCTC-3' |
| Nanog forward | 5'-TCCTCCTCTTCCTCTATACTAAC-3' |
| Nanog reverse | 5'-CCCACAATCACAGGCATAG-3' |
| SOX2 forward | 5'-TACAGCATGTCCTACTCGCAG-3' |
| SOX2 reverse | 5'-GAGGAAGAGGTAACCACAGGG-3' |
| CNND1 forward | 5'-AGAGGCGGAGGAGAACAAAC-3' |
| CNND1 reverse | 5'-GGCGGATTGGAAATGAACTT-3' |
| c-Myc forward | 5'-TCCCTCCACTCGGAAGGAC-3' |
| c-Myc reverse | 5'-CTGGTGCATTTTCGGTTGTTG-3' |
| MMP7 forward | 5'-GAGTGAGCTACAGTGGGAACA-3' |
| MMP7 reverse | 5'-CTATGACGCGGGAGTTTAACAT-3' |
| c-Jun forward | 5'-TCCAAGTGCCGAAAAAGGAAG-3' |
| c-Jun reverse | 5'-CGAGTTCTGAGCTTTCAAGGT-3' |
| CD44 forward | 5'-CTGCCGCTTTGCAGGTGTA-3' |
| CD44 reverse | 5'-CATTGTGGGCAAGGTGCTATT-3' |
| CD24 forward | 5'-CTCCTACCCACGCAGATTTATTC-3' |
| CD24 reverse | 5'-AGAGTGAGACCACGAAGAGAC-3' |
| CTNNB1 forward | 5'-AGCTTCCAGACACGCTATCAT-3' |
| CTNNB1 reverse | 5'-CGGTACAACGAGCTGTTTCTAC-3' |
| AXIN1 forward | 5'-GACCTGGGGTATGAGCCTGA-3' |
| AXIN1 reverse | 5'-GGCTTATCCCATCTTGGTCATC-3' |
| APC forward | 5'-AAGCATGAAACCGGCTCACAT-3' |
| APC reverse | 5'-CATTCGTGTAGTTGAACCCTGA-3' |
| CK1 forward | 5'-AGTGGCAGTGAAGCTAGAATCT-3' |
| CK1 reverse | 5'-CGCCCAATACCCATTAGGAAGTT-3' |
| GSK3β forward | 5'-GGCAGCATGAAAGTTAGCAGA-3' |
| GSK3β reverse | 5'-GGCGACCAGTTCTCCTGAATC-3' |
| RBM5 forward | 5′-GCACGACTATAGGCATGACAT-3′ |
| RBM5 reverse | 5′-AGTCAAACTTGTCTGCTCCA-3′ |
| RBM6 forward | 5′-GCGGGGCACATATGATTTAG-3′ |
| RBM6 reverse | 5′-TTCCTAAAATCAGAACGAGACTGA-3′ |
| **Sequences of primers used for plasmid construction** | |
| pcDNA3.1-RBM5-AS1 forward | 5'-CGGGATCCAAATGCCGCCACAGACTTTCACTAT-3' |
| pcDNA3.1-RBM5-AS1 reverse | 5'-CCCTCGAGCAGAAGAATCGCTTGAATCCAGGAG-3' |
| pcDNA3.1-RUNX2 forward | 5'-CGGCTAGCATGCGTATTCCCGTAGATCCGAGC-3' |
| pcDNA3.1-RUNX2 reverse | 5'-CCCTCGAGTCAATATGGTCGCCAAACAGAT-3' |
| pGL3-Basic-RBM5-AS1 pro-WT forward | 5'-ATTTCTCTATCGATAGGTACCGGTAGCAATAATAGAATTAAA  TGGACAAC-3' |
|  |  |
| pGL3-Basic-RBM5-AS1 pro-WT reverse | 5'-CAGATCTCGAGCCCGGGCTAGCTTATAAGTAATTCCTTATTTC  TGCCTGAA-3' |
|  |  |
| pGL3-Basic-RBM5-AS1 pro-MUT (RUNX2) forward | 5'-AGGCTTGTGTTGAGGCAGGCAGAAATAAGGAATTACTTATAA  GC-3' |
| pGL3-Basic-RBM5-AS1 pro-MUT (RUNX2) reverse | 5'-TGCCTCAACACAAGCCTCCTGGAGGCGTAACTGCTCA-3' |
| **Sequences of primers used for ChIP-qPCR** |  |
| P1 forward | 5'-GAGGACCAACCACCGCCACCTA-3' |
| P1 reverse | 5'-CAGCACTCCCTGACTCCGTTTACC-3 |
| P2 forward | 5'-GAGTATACCTAGCACCTACCCCTAAA-3' |
| P2 reverse | 5'-CCCACCCTTGATCCTCCCAC-3 |
| P3 forward | 5'-ACACCACTCCCAGGCTAATCTC-3' |
| P3 reverse | 5'-TCTGCCTGAAGACCACAAAGC-3' |

| **Supplementary Table S3. Antibodies used for western blotting (WB), immunoprecipitation (IP), and flow cytometry (FC).** | | | | | |
| --- | --- | --- | --- | --- | --- |
| **Protein** | **Applications** | **Antibody** | **Origin** | **dilution** | **Molecular weight** |
| GAPDH | WB | D16H11, Cell Signaling Technology | Rabbit | 1:1000 | 37 KD |
| Sox2 | WB | sc-365964, Santa cruz | Mouse | 1:500 | 34KD |
| OCT4 | WB | sc-101534, Santa cruz | Mouse | 1:500 | 52KD |
| Nanog | WB | sc-293121, Santa cruz | Mouse | 1:500 | 40KD |
| ALDH1A1 | WB | sc-374076, Santa cruz | Mouse | 1:500 | 56KD |
| E-cadherin | WB | 3195, Cell Signaling Technology | Rabbit | 1:1000 | 135KD |
| Vimentin | WB | ab92547, Abcam | Rabbit | 1:1000 | 54KD |
| Zeb1 | WB | 3396, Cell Signaling Technology | Rabbit | 1:1000 | 200KD |
| Slug | WB | 9585, Cell Signaling Technology | Rabbit | 1:1000 | 30KD |
| Twist | WB | 46702, Cell Signaling Technology | Rabbit | 1:1000 | 26KD |
| Snail | WB | 3879, Cell Signaling Technology | Rabbit | 1:1000 | 29KD |
| CD44 | WB | 3570, Cell Signaling Technology | Mouse | 1:1000 | 80KD |
| c-Myc | WB | 5605, Cell Signaling Technology | Rabbit | 1:1000 | 57-65KD |
| c-Jun | WB | 9165, Cell Signaling Technology | Rabbit | 1:1000 | 43KD |
| MMP7 | WB | 71031, Cell Signaling Technology | Rabbit | 1:1000 | 20KD |
| Cyclin D1 | WB | 2978, Cell Signaling Technology | Rabbit | 1:1000 | 36KD |
| TCF4 | WB | 2565, Cell Signaling Technology | Rabbit | 1:1000 | 58KD |
| β-catenin | WB, IF, IP | 8480, Cell Signaling Technology | Rabbit | 1:1000, 1:100, 1:25 | 92KD |
| Histone H3 | WB | 4499, Cell Signaling Technology | Rabbit | 1:1000 | 17KD |
| p-β-catenin | WB | Wl03554，Wanlei | Rabbit | 1:1000 | 92KD |
| Axin1 | WB | 16541-1-AP, proteintech | Rabbit | 1:1000 | 110KD |
| GSK3β | WB | 5676, Cell Signaling Technology | Rabbit | 1:1000 | 47KD |
| CK1 | WB | Wl04799，Wanlei | Rabbit | 1:1000 | 47KD |
| RBM5 | WB | 19930-1-AP, Proteintech | Rabbit | 1:1000 | 110KD |
| RBM6 | WB | 14360-1-AP, Proteintech | Rabbit | 1:1000 | 129KD |
| IgG | CHIP, RIP | ab18413, Abcam | Mouse | 1:10 | 150KD |
| RUNX2 | CHIP | EPR22858-106, Abcam | Rabbit | 1:1000 | 55KD |
| CD44 | FC | 559942, BD Pharmingen™ | Mouse | 1:200 | 81KD |
| CD24 | FC | 555428, BD Pharmingen™ | Mouse | 1:200 | 9KD |
